# Genetic identification and characterization of novel loci for flag leaf morphology traits in Chinese endemic wheat

**DOI:** 10.1002/tpg2.70245

**Published:** 2026-04-28

**Authors:** Md Nahibuzzaman Lohani, Longxing Su, Lu Lu, Li Yin, Yanlin Liu, Qiang Xu, Yunfeng Jiang, Qiantao Jiang, Guoyue Chen, Yuming Wei, Chunji Liu, Quan Xie, Jian Ma

**Affiliations:** ^1^ State Key Laboratory of Crop Gene Exploration and Utilization in Southwest China/Triticeae Research Institute Sichuan Agricultural University Chengdu China; ^2^ College of Culinary and Food Science Engineering Sichuan Tourism University Chengdu Sichuan China; ^3^ School of Agriculture Henan Institute of Science and Technology Xinxiang China; ^4^ State Key Laboratory of Crop Genetics & Germplasm Enhancement and Utilization Nanjing Agricultural University Nanjing China

## Abstract

Flag leaf morphology (FLM) is a critical determinant of wheat (*Triticum aestivum* L.) photosynthetic efficiency and grain yield. Chinese endemic wheat, a unique genetic reservoir formed by long‐term adaptation to diverse and heterogeneous environments in China, harbors abundant, untapped allelic variation for traits related to environmental resilience and yield architecture. Mining this distinctive germplasm is therefore essential for identifying novel genetic determinants underlying complex traits like FLM. In this study, a genome‐wide association study was conducted on 182 Chinese endemic wheat accessions using 38,490 single‐nucleotide polymorphisms. We identified four stable quantitative trait loci (QTL) associated with FLM traits, three of which for flag leaf width (*QFLW.sau.5A*, *QFLW.sau.5B*, and *QFLW.sau.5D*) were intriguingly located on the homoeologous chromosomes of group 5, suggesting the action of a conserved, multi‐subgenome genetic system. None of these QTLs overlapped with previously reported loci, highlighting their novelty and the value of this endemic gene pool. Pleiotropic analysis revealed that these loci also influence plant height and spike architecture. Their breeding value was further confirmed by validation in an independent population. Thirteen high‐confidence candidate genes with elevated expression in flag leaf tissue were predicted, many involved in leaf development, photosynthesis, and stress responses. Collectively, by mining the unique genetic diversity of Chinese endemic wheat, we have uncovered a novel, homoeologous QTL system and candidate genes for FLM. These findings provide both a conceptual advance in understanding the polyploid genetic architecture of this key trait and valuable functional targets/molecular markers for improving photosynthetic efficiency and yield potential in wheat breeding.

## Plain Language Summary

Flag leaf shape is a key factor in wheat yield, influencing how much sunlight the plant captures. Chinese endemic wheat, a unique group adapted to local conditions, holds valuable genetic diversity for this trait. In this study, we analyzed 182 of these wheat types and identified four new genetic regions linked to flag leaf width and shape. Three of these regions are located on homoeologous positions of different chromosomes, suggesting a coordinated genetic system. These findings were confirmed in a separate wheat population. We also predicted 13 candidate genes likely involved in flag leaf development. This research provides new genetic markers and highlights useful wheat varieties for breeding programs aiming to develop higher‐yielding wheat with better leaf architecture.

**Abbreviations**
AD: anthesis date
BLUP: best linear unbiased prediction
ETN: effective tiller number
FLA: flag leaf area
FLANG: flag leaf angle
FLL: flag leaf length
FLM: flag leaf morphology
FLR: flag leaf ratio
FLW: flag leaf width
GWAS: genome‐wide association study
LD: linkage disequilibrium; LOD, logarithm of the odds;
PH: plant height
QTL: quantitative trait locus
SL: spike length
SNP: single‐nucleotide polymorphism
SNS: spikelet number per spike

## 1 INTRODUCTION

Wheat (*Triticum aestivum* L.) is a cornerstone of global food security, serving as a staple crop for approximately 35% of the world's population. It contributes about 19% of total caloric intake and is the leading plant‐derived protein source, providing around 21% of dietary protein worldwide (Grote et al., 2021; Poutanen et al., 2022; Tadesse et al., 2019). With the global population projected to approach 10 billion by 2050 and the availability of arable land steadily declining, increasing wheat yield is an urgent priority (Kumar et al., 2024). Achieving this will require not only improved agronomic practices but also accelerated genetic gains beyond current rates (Hall & Richards, 2013).

Photosynthetic capacity has long been recognized as a major driver of crop yield potential (Ort et al., 2015; Zhu et al., 2008). In wheat, the flag leaf is the primary photosynthetic organ during the reproductive stage, contributing 40%–60% of total photosynthesis and supplying over 40% of assimilates during grain filling (Ba et al., 2020; Du et al., 2022; Duncan, 1971; Khaliq et al., 2008; Sharma et al., 2003). Flag leaf morphology (FLM) including length, width, area, and angle directly affects canopy structure, light interception, and ultimately grain yield potential (Duncan, 1971; Guitman et al., 1991; Sakamoto et al., 2006; Sharma et al., 2003). Understanding the genetic architecture underlying these traits is therefore crucial for breeding high‐yielding wheat cultivars (J. Ma et al., 2020).

Flag leaf‐related traits are typically quantitative in nature, controlled by multiple genes or quantitative trait loci (QTL) and strongly influenced by environmental conditions (Coleman et al., 2001; Kobayashi et al., 2003; Simón, 1999). Over the past two decades, numerous QTLs for flag leaf length (FLL), flag leaf width (FLW), flag leaf area (FLA), and flag leaf angle (FLANG) have been detected across the A, B, and D subgenomes of wheat (Hussain et al., 2017; K. Liu et al., 2018; Y. Liu et al., 2017). Notable examples include *QFll.cib‐2B.2*, *qFll‐4B.1*, *QFll.sicau‐2D.3*, and *QFll.sicau‐5B.3* for FLL (L. Chen et al., 2022; Fan et al., 2015; J. Ma et al., 2020); *QFlw.sicau‐2D*, *QFlw‐4B*, *QFlw‐5B*, and *QFlw‐6B* for FLW (Fan et al., 2015; J. Ma et al., 2020; Zhao et al., 2022); and *QFla.cib‐2B.2*, *QFla.sicau‐2D*, *qFla‐4B.1*, q*Fla‐5B*, and *qFla‐6B.2* for FLA (L. Chen et al., 2022; Fan et al., 2015; J. Ma et al., 2020). In addition, *QFlr.sicau‐5B* and *QFlr.cau‐5A.2* have been reported for flag leaf ratio (FLR) (J. Ma et al., 2020; Wu et al., 2016). Functional studies have also identified promising candidate genes, such as *TaWOX5*, which increases FLW in transgenic wheat (K. Wang, Shi, et al., 2022), and the recessive genes *TaFLA1* and *TaSPL8*, which act synergistically to regulate FLANG (Q. Wang et al., 2024).

Despite recent advances, the discovery of stable and novel loci regulating FLM traits across environments remains limited, particularly within diverse natural wheat populations. Chinese endemic wheat, which originated from primitive hexaploid forms through long‐term natural and artificial selection across heterogeneous ecological regions (Dong et al., 1981), harbors abundant genetic variation in both morphological and agronomic traits (X. Chen, 1980; Huang et al., 2002; Shao et al., 1981; Z. Wang et al., 2010). These subspecies also possess adaptive advantages, including stress tolerance and disease resistance (Guo et al., 2001; Yang et al., 2011). Due to their diversity and cross‐compatibility with bread wheat, they constitute a critical genetic reservoir for wheat improvement (J. Li et al., 2020).

Consequently, Chinese endemic wheat provides an ideal population for genome‐wide association studies (GWAS) aimed at uncovering alleles controlling flag leaf variation and its contribution to yield. GWAS, especially when coupled with high‐density single‐nucleotide polymorphism (SNP) genotyping platforms, has proven highly effective in dissecting complex traits in wheat (Ye et al., 2019). By leveraging linkage disequilibrium (LD) in diverse germplasm, GWAS enables the detection of marker–trait associations with high resolution, thereby offering valuable targets for marker‐assisted selection.

In this study, we employed a 55K SNP array to conduct GWAS on 182 Chinese endemic wheat accessions evaluated for FLM traits over four growing seasons. Our analysis identified four stable and potentially novel QTL associated with key morphological traits, along with 13 putative candidate genes exhibiting high expression in flag leaf tissues throughout the growth cycle and during grain development. These findings offer new insights into the genetic mechanisms underlying light capture and photosynthetic assimilation, and provide valuable molecular resources for improving grain yield through marker‐assisted breeding in wheat.

## 2 MATERIALS AND METHODS

### 2.1 Plant materials

A total of 182 accessions of Chinese endemic wheat were used in this study, obtained from the Triticeae Germplasm Resources Bank of Sichuan Agricultural University and the Chinese Crop Germplasm Resources Bank (J. Li et al., 2020). This collection comprised 109 Tibetan semi‐wild wheat accessions, 61 Yunnan hulled wheat accessions, and 12 Xinjiang rice wheat accessions (Table S1). Furthermore, a validation population comprising 220 Sichuan wheat accessions, including both cultivars and landraces, was used in this study (Lohani et al., 2025). Compared with the GWAS panel, which mainly comprised Chinese endemic wheat, this population represents a broader spectrum of cultivated germplasm, making it suitable for evaluating the robustness and transferability of the identified QTL (Lohani et al., 2025).

### 2.2 Phenotypic assessment of FLM traits

Field experiments were conducted at Chongzhou (30°33′37.3″ N, 103°38′45.4″ E) in 2022, 2023, and 2025, and at Wenjiang (30°43′ N, 103°51′ E) in 2025, hereafter denoted as 2022CZ, 2023CZ, 2025CZ, and 2025WJ. Each accession was sown in a single 2‐m row, with 0.1 m plant spacing and 0.3‐m row spacing (J. Ma et al., 2020). Fertilization consisted of superphosphate (80 kg/ha) and nitrogen (100 kg/ha) applied at sowing. Field management followed local agronomic practices (Q. Liu et al., in press; Zhang et al., 2024; J. Zhou et al., 2024).

Flag leaf traits were recorded after anthesis on healthy leaves from at least eight individual plants per line, with one representative tiller sampled per plant to avoid repeated measurements from the same genetic unit. FLL was measured manually using a ruler as the distance from the base to the tip of the leaf, and FLW as the maximum width of the leaf blade (J. Ma et al., 2020). FLA was estimated as FLL × FLW × 0.75, while FLR was calculated as FLL/FLW (J. Ma et al., 2020; Tu et al., 2021).

### 2.3 Phenotypic data collection and analysis

For each line, measurements from a minimum of eight plants were averaged to obtain trait values. FLM traits (FLL, FLW, FLA, and FLR) were assessed across four environments: 2022CZ, 2023CZ, 2025CZ, and 2025WJ. Additional agronomic traits, including plant height (PH), effective tiller number (ETN), spike length (SL), and spikelet number per spike (SNS), were measured under the same environments, while anthesis date (AD) was recorded only at 2025WJ.

Best linear unbiased prediction (BLUP) for the four flag leaf traits across environments was estimated using SAS v8.0 (SAS Institute). Broad‐sense heritability (*H* ^2^) was calculated across environments following the formula *H* ^2^ = *V*_G_/(*V*_G_ + *V*_E_), where *V*_G_ denotes genotypic variance and *V*_E_ represents environmental variance (Smith et al., 1998). Estimates of *V*_G_ and *V*_E_ were derived from the BLUP model.

For each environment and for the BLUP dataset, the maximum and minimum values of all traits were recorded. Descriptive statistics, including mean, standard deviation, and coefficient of variation, were computed. Trait correlations were analyzed and visualized in R using the ggplot2 package (Ginestet, 2011), while boxplots were generated in GraphPad Prism v9.5.1 (GraphPad Software).

### 2.4 Genotyping analysis

Leaf tissues were collected from 2‐week‐old seedlings, and genomic DNA was extracted using a modified cetyltrimethylammonium bromide protocol (Saghai‐Maroof et al., 1984). In a previous study, J. Li et al. (2020) genotyped 213 accessions of Chinese endemic wheat using the wheat 55K SNP array (Affymetrix Axiom Wheat55K) at CapitalBio Technology. Markers with ≤5% missing data and a minor allele frequency ≥5% were retained for downstream genetic analyses (J. Li et al., 2020). For the present study, genotyping data for 182 accessions were retrieved. Genotyping of the validation population followed the methodology described by Ye et al. (2019).

### 2.5 Population structure, kinship, and LD analysis

Population structure (*Q*‐matrix) was inferred using a Bayesian model‐based clustering approach (Pritchard et al., 2000) in STRUCTURE v2.3.4. Five independent runs were conducted under an admixture model with *K* values from 1 to 10, a burn‐in of 100,000 iterations, and 100,000 MCMC (Markov Chain Monte Carlo) replicates. The optimal *K* was determined using the Δ*K* method in STRUCTURE SELECTOR (Y. L. Li & Liu, 2018). Kinship (*K*‐matrix) between accessions was estimated in TASSEL v5.2.38 based on the identity‐by‐state (IBS) method, and pairwise LD was calculated as the squared correlation of allele frequencies (*r* ^2^) between intrachromosomal markers with known positions (Ye et al., 2019). Loci within the same LD half‐decay distance on a chromosome were considered part of the same QTL block, integrating population structure, relatedness, and LD patterns to identify high‐confidence (HC) candidate loci (J. Li et al., 2020; Lohani et al., 2025; Ye et al., 2019).

### 2.6 Genome‐wide association analysis

GWAS was conducted on 182 wheat accessions to identify loci controlling flag leaf traits. Analyses were performed using the rMVP package (Yin et al., 2021) in R with a mixed linear model (MLM) framework (Yu et al., 2006), incorporating both *Q* and *K* matrices to account for potential confounding effects. The dataset comprised 38,490 high‐quality SNP markers with known genomic positions (J. Li et al., 2020).

A stringent significance threshold of *p* ≤ 0.001 (−log_10_ *p* ≥ 3) was applied to select SNPs with strong statistical support and reduce the likelihood of false positives. To further ensure robustness, only SNPs consistently detected across two or more independent test environments and BLUP datasets were considered HC associations. This dual criterion of stringent *p*‐value and reproducibility across environments ensures that identified loci represent reliable genetic signals and were subsequently prioritized for validation and functional characterization.

### 2.7 Candidate gene prediction

Candidate gene prediction was performed by extracting genes located within ±2.5 Mb of the most significant SNPs for each QTL using the WheatOmics database (S. Ma et al., 2021), based on the estimated LD half‐decay distance. Genes within these intervals were retrieved from the reference genome annotation and subjected to a multistep prioritization process. First, genes with detectable expression in flag leaf tissues were retained based on transcriptomic data from WheatOmics. Second, candidates were further prioritized based on (i) proximity to the peak SNP; (ii) relatively higher expression levels in flag leaf tissue compared with other organs; and (iii) functional annotation related to plant growth, leaf development, photosynthesis, or stress response.

## 3 RESULTS

### 3.1 Phenotypic variation and heritability

**FIGURE 1:**
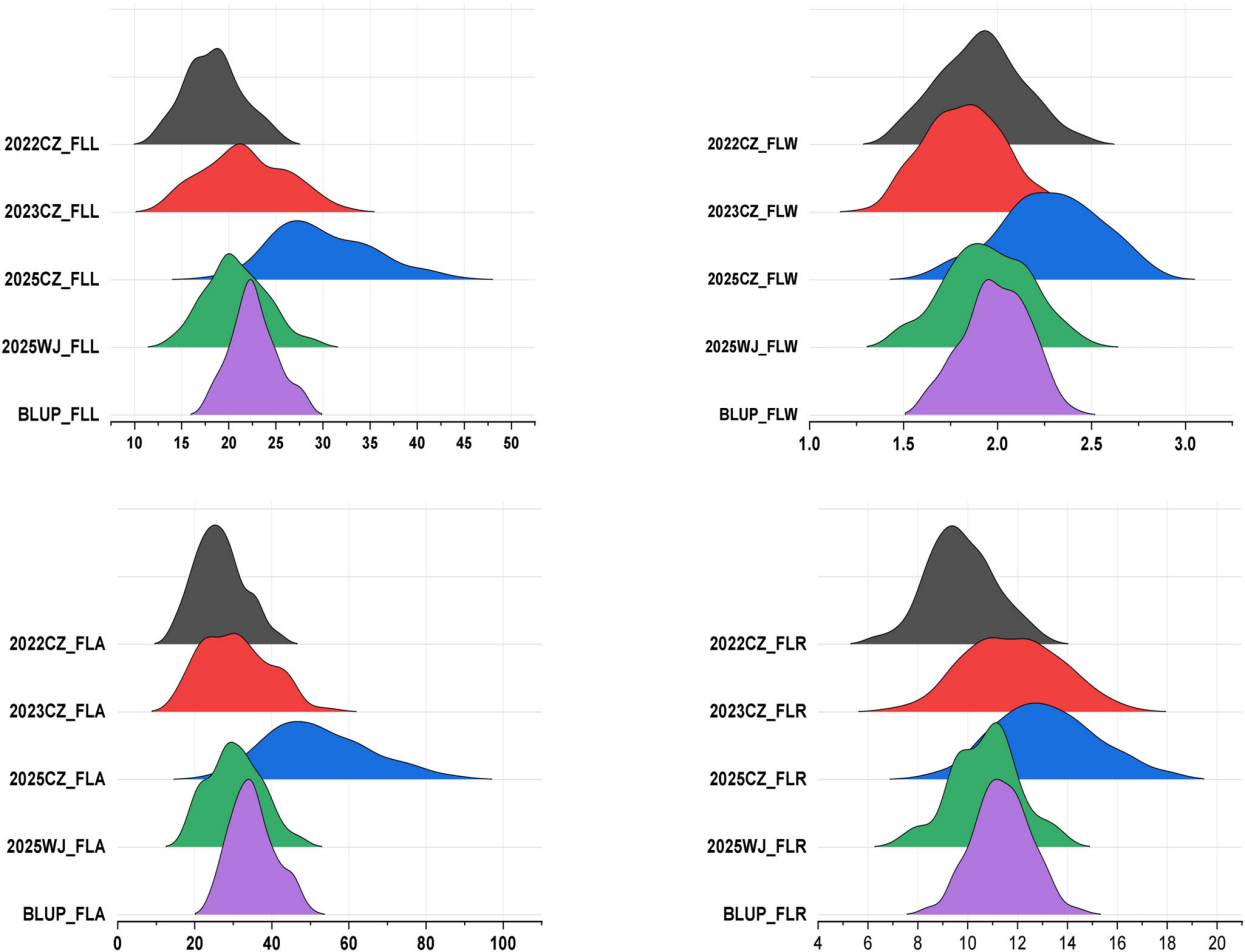
Density plot showing phenotypic distribution of various flag leaf morphology (FLM) traits in 182 accessions of Chinese endemic wheat in four different environments (2022CZ, 2023CZ, 2025CZ, and 2025WJ) and best linear unbiased prediction (BLUP) values. FLA, flag leaf area; FLL, flag leaf length; FLR, flag leaf ratio; FLW, flag leaf width.

In all the tested environments, FLM showed significant variations (Table 1). The distribution of FLM traits was consistent in all the tested environments except 2025CZ, with slightly higher measurements for all FLM traits (Figure 1). For FLL, the range varied from 11.92 to 44.70 cm, with the mean values differing across environments and years, and a heritability of 86.23%. FLW exhibited a range from 1.30 to 2.88 cm, with a heritability of 91.65%. FLA had the broadest range, from 13.68 to 88.69 cm^2^, and a heritability of 86.22%. FLR varied from 6.19 to 18.18, with a heritability of 88.97%. The BLUP values provided a more stable estimate of these traits, with FLL ranging from 17.55 to 28.34 cm, FLW from 1.62 to 2.41 cm, FLA from 23.80 to 50.06 cm^2^, and FLR from 8.31 to 14.60 (Table 1). These findings highlight the significant impact of both genetic and environmental factors on FLM, with high heritability values indicating a strong genetic influence on these traits.

**TABLE 1:** Phenotypic assessment of 182 accessions of Chinese endemic wheat for flag leaf morphology (FLM) traits at multiple environments and best linear unbiased prediction (BLUP) datasets.

Trait	Environment	Min	Max	Mean	STDEV	CV (%)	*H* ^2^ (%)
FLL (cm)	2022CZ	11.92	25.63	18.44	3.01	16.31	86.23
	2023CZ	13.01	32.62	21.80	4.40	20.20	
	2025CZ	17.37	44.70	29.95	5.24	17.51	
	2025WJ	13.53	29.50	20.84	3.24	15.54	
	BLUP	17.55	28.34	22.65	2.41	10.64	
FLW (cm)	2022CZ	1.43	2.48	1.91	0.22	11.61	91.65
	2023CZ	1.30	2.36	1.83	0.21	11.60	
	2025CZ	1.60	2.88	2.28	0.27	11.67	
	2025WJ	1.45	2.50	1.95	0.22	11.45	
	BLUP	1.62	2.41	1.99	0.17	8.62	
FLA (cm^2^)	2022CZ	13.68	42.57	26.40	6.24	23.65	86.22
	2023CZ	14.56	56.26	30.63	8.77	28.63	
	2025CZ	23.09	88.69	52.06	13.25	25.46	
	2025WJ	17.02	48.58	30.53	6.91	22.63	
	BLUP	23.80	50.06	34.98	5.75	16.43	
FLR	2022CZ	6.19	13.16	9.74	1.36	13.96	88.97
	2023CZ	6.87	16.71	11.82	1.91	16.13	
	2025CZ	8.18	18.18	13.14	2.02	15.39	
	2025WJ	7.15	14.03	10.72	1.36	12.68	
	BLUP	8.31	14.60	11.38	1.15	10.07	

Abbreviations: 2022CZ, Chongzhou 2022; 2023CZ, Chongzhou 2023; 2025CZ, Chongzhou 2025; 2025WJ, Wenjiang 2025; CV, coefficient of variation; FLA, flag leaf area; FLL, flag leaf length; FLR, flag leaf ratio; FLW, flag leaf width; *H* ^2^, broad sense heritability; STDEV, standard deviation.

### 3.2 Correlation analysis between FLM and agronomic traits

The correlation analysis revealed significant relationships between leaf traits across different environments (Table 2). For FLL, correlations ranged from 0.384 to 0.609 (*p* < 0.01), with the strongest correlation between 2023CZ and 2025WJ. FLW showed correlations from 0.520 to 0.684 (*p* < 0.01), with the highest correlation between 2023CZ and 2025WJ. FLA had correlations from 0.442 to 0.688 (*p* < 0.01), with the strongest correlation between 2023CZ and 2025WJ. FLR exhibited correlations from 0.418 to 0.691 (*p* < 0.01), with the highest correlation between 2022CZ and 2023CZ. The BLUP values showed high correlations with other environments, ranging from 0.738 to 0.884 (*p* < 0.01).

**TABLE 2:** Correlation coefficients of flag leaf‐related traits in different environments.

Trait	Environment	2022CZ	2023CZ	2025CZ	2025WJ	BLUP
FLL (cm)	2022CZ	1				
	2023CZ	0.606^**^	1			
	2025CZ	0.426^**^	0.384^**^	1		
	2025WJ	0.609^**^	0.602^**^	0.605^**^	1	
	BLUP	0.766^**^	0.783^**^	0.784^**^	0.844^**^	1
FLW (cm)	2022CZ	1				
	2023CZ	0.595^**^	1			
	2025CZ	0.520^**^	0.637^**^	1		
	2025WJ	0.641^**^	0.680^**^	0.684^**^	1	
	BLUP	0.807^**^	0.853^**^	0.855^**^	0.884^**^	1
FLA (cm^2^)	2022CZ	1				
	2023CZ	0.543^**^	1			
	2025CZ	0.442^**^	0.494^**^	1		
	2025WJ	0.583^**^	0.588^**^	0.623^**^	1	
	BLUP	0.738^**^	0.797^**^	0.863^**^	0.837^**^	1
FLR	2022CZ	1				
	2023CZ	0.691^**^	1			
	2025CZ	0.433^**^	0.418^**^	1		
	2025WJ	0.663^**^	0.622^**^	0.544^**^	1	
	BLUP	0.828^**^	0.841^**^	0.782^**^	0.851^**^	1

Abbreviations: 2022CZ, Chongzhou 2022; 2023CZ, Chongzhou 2023; 2025CZ, Chongzhou 2025; 2025WJ, Wenjiang 2025; BLUP, best linear unbiased prediction; FLA, flag leaf area; FLL, flag leaf length; FLR, flag leaf ratio; FLW, flag leaf width.

** denotes that correlation is significant at the 0.01 level (two‐tailed).

**FIGURE 2:**
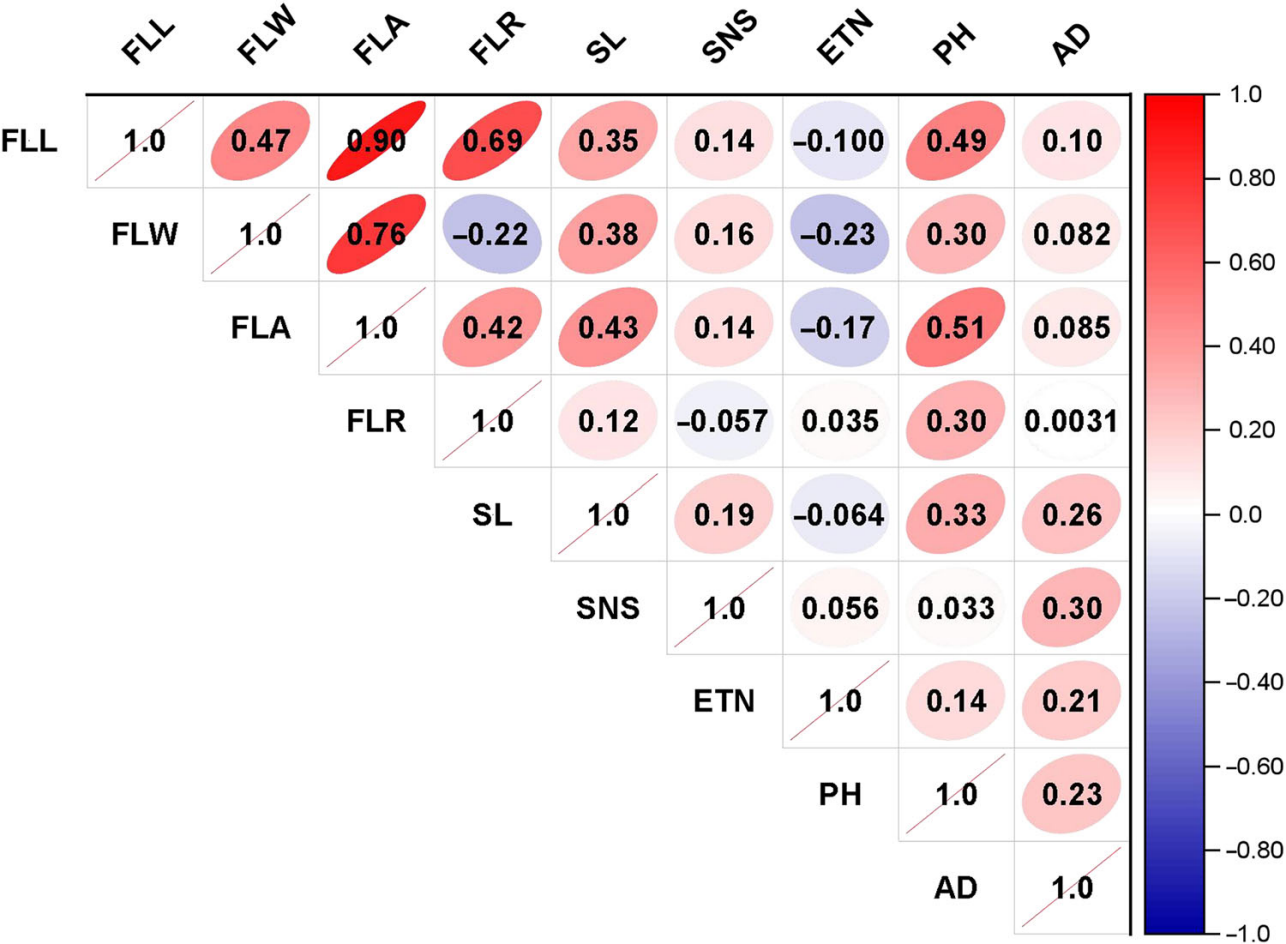
Correlation matrix for four flag leaf morphology (FLM) traits and other agronomic traits. AD, anthesis date; ETN, effective tiller number; FLA, flag leaf area; FLL, flag leaf length; FLR, flag leaf ratio; FLW, flag leaf width; PH, plant height; SL, spike length; SNS, spikelet number per spike.

Significant positive correlations were observed among FLM traits, with the strongest between FLL and FLA (*r* ≈ 0.90, *p* < 0.01). FLL was positively associated with both FLW and FLR, while FLW showed a negative correlation with FLR. Among agronomic traits, PH and SL were positively correlated with several FLM traits, particularly FLA, whereas ETN and SNS displayed negative or little associations (Figure 2). AD showed weak or nonsignificant correlations, suggesting minimal influence of phenology on FLM.

Overall, these results indicate that FLM traits are not only highly interrelated but also strongly associated with important agronomic traits, particularly PH and SL, underscoring their potential role in yield‐related trait improvement.

### 3.3 Population structure, principal component analysis, and LD

Structure analysis indicated that the 182 wheat accessions could be grouped into two subpopulations (*K* = 2; Figure S1A), consistent with previous findings (J. Li et al., 2020). However, the inferred clusters did not strictly correspond to the predefined subspecies categories (Tibetan semi‐wild, Yunnan hulled, and Xinjiang rice wheat), as each subgroup contained a mixture of these types.

Principal component analysis further supported this observation, with the first three principal components (PC1 = 21.52%, PC2 = 9.52%, and PC3 = 6.47%) explaining a substantial proportion of the genetic variation (Figure S1B). The continuous distribution of accessions without clear separation indicates weak population stratification within the panel. Collectively, these results suggest that the genetic structure is relatively weak and unlikely to strongly confound downstream association analyses.

The LD half‐decay distance, estimated based on *r* ^2^ values between intrachromosomal SNP pairs, was 2.58 Mb (Figure S1C), consistent with the previously reported value of 2.57 Mb (J. Li et al., 2020). Accordingly, loci located within 2.5 Mb on the same chromosome were considered to belong to the same QTL block.

### 3.4 Genome‐wide association study

**FIGURE 3:**
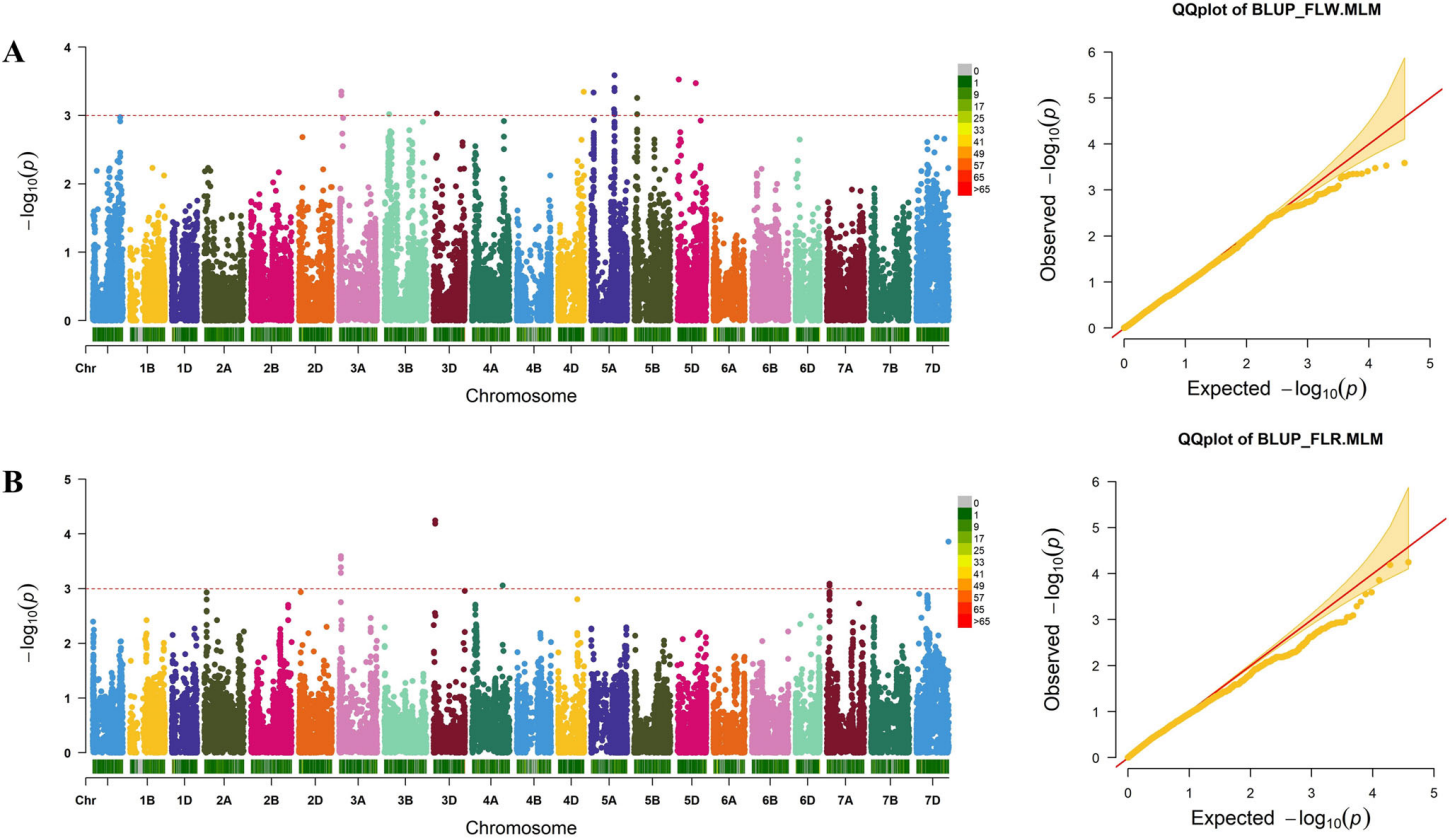
Manhattan and quantile–quantile (*Q*–*Q*) plots showing the results of genome‐wide association studies (GWASs) for (A) flag leaf width (FLW) and (B) flag leaf ratio (FLR) using the *Q* + *K*, mixed linear model (MLM) method in best linear unbiased prediction (BLUP) datasets. The positions of single‐nucleotide polymorphism (SNP) on chromosomes are indicated on the *x*‐axis relative to their −log_10_(*p*) values on the *y*‐axis. The red dotted line refers to the threshold of −log10(*p*) = 3.

A total of 38,490 high‐quality SNPs were used to perform GWAS using MLM, *Q* + *K* as covariates, applying a significance threshold of *p* ≤ 0.001 (−log10 *p* ≥ 3) (Figure 3). Analyses were conducted across multiple environments (2022CZ, 2023CZ, 2025CZ, and 2025WJ) to ensure robust detection of stable loci.

Quantile–quantile (*Q*–*Q*) plots for all traits showed good agreement between observed and expected *p*‐values. The genomic inflation factors (λGC), calculated from BLUP‐based GWAS results, ranged from 0.99 to 1.11, with values of 1.014 (FLL), 0.992 (FLW), 1.072 (FLA), and 1.113 (FLR). These results indicate overall effective control of population structure and relatedness, although a slightly elevated λGC for FLR suggests minor residual inflation. Despite the detection of significant associations in the Manhattan plots, the *Q*–*Q* plots exhibited only slight deviation from the expected distribution at the extreme tail, likely reflecting the moderate effect sizes of the detected loci and the effective control of confounding factors by the *Q* + *K* model.

Five significant SNPs were detected on chromosomes 3D, 5A, 5B, and 5D for different FLM traits. Based on LD half‐decay distances, these SNPs were consolidated into four major and stable QTL (Table 3). For FLW, three QTL were identified, intriguingly located one each on the homoeologous chromosomes 5A, 5B, and 5D. *QFLW.sau.5B*, associated with AX‐110931135 at 56.95 Mb, was detected consistently in 2023CZ, 2025CZ, and BLUP datasets, with LOD (logarithm of the odds) scores up to 3.46 and phenotypic variance explained (*R* ^2^) ranging from 5.92% to 6.66%. *QFLW.sau.5A* (AX‐109378245 at ∼458.99 Mb) and *QFLW.sau.5D* (AX‐110979994 at ∼358 Mb) were detected in 2022CZ, 2023CZ, and BLUP environments. LOD scores for *QFLW.sau.5A* ranged from 3.22 to 3.59 (*R* ^2^ 4.60%–5.28%), whereas *QFLW.sau.5D* exhibited LOD scores from 3.47 to 3.69 (*R* ^2^ 5.05%–5.44%). No significant SNPs were detected for FLL and FLA. However, one QTL for FLR, *QFLR.sau.3D*, associated with AX‐109854359 and AX‐95207852 at ∼30 Mb, was identified in three environments (2022CZ, 2025WJ, and BLUP), with LOD scores ranging from 3.61 to 4.90 and *R* ^2^ between 4.55% and 6.52% (Table 3).

**TABLE 3:** List of identified quantitative trait locus (QTL) for flag leaf morphology (FLM) traits at multiple environments and best linear unbiased prediction (BLUP) datasets.

Trait	QTL	SNP	Chrom	Position	Allele	LOD	Marker *R* ^2^ (%)	Environments
FLW	*QFLW.sau.5A*	AX‐109378245	5A	458996188	T/C	3.22–3.59	4.60–5.28	2022CZ, 2023CZ, BLUP
	*QFLW.sau.5B*	AX‐110931135	5B	56954562	G/T	3.14–3.46	5.92–6.66	2023CZ, 2025CZ, BLUP
	*QFLW.sau.5D*	AX‐110979994	5D	358012495	G/A	3.47–3.69	5.05–5.44	2022CZ, 2023CZ, BLUP
FLR	*QFLR.sau.3D*	AX‐109854359	3D	30233478	T/C	3.61–4.88	4.55–6.52	2022CZ, 2025WJ, BLUP
		AX‐95207852	3D	30085322	G/C	3.65–4.90	4.56–6.49	2022CZ, 2025WJ, BLUP

Abbreviations: 2022CZ, Chongzhou 2022; 2023CZ, Chongzhou 2023; 2025CZ, Chongzhou 2025; 2025WJ, Wenjiang 2025; FLR, flag leaf ratio; FLW, flag leaf width; SNP, single‐nucleotide polymorphism.

To our best knowledge, these four QTL have not been reported previously and are therefore considered potentially novel. The identification of these loci, together with candidate genes based on LD intervals, provides valuable insights into the genetic determinants of flag leaf traits and offers practical targets for wheat breeding and crop improvement.

### 3.5 Effects of the identified QTL

We further evaluated the effects of the identified QTL for FLW and FLR on key agronomic traits, including SL, SNS, ETN, AD, and PH, using BLUP‐derived phenotypic values to minimize environmental noise.

**FIGURE 4:**
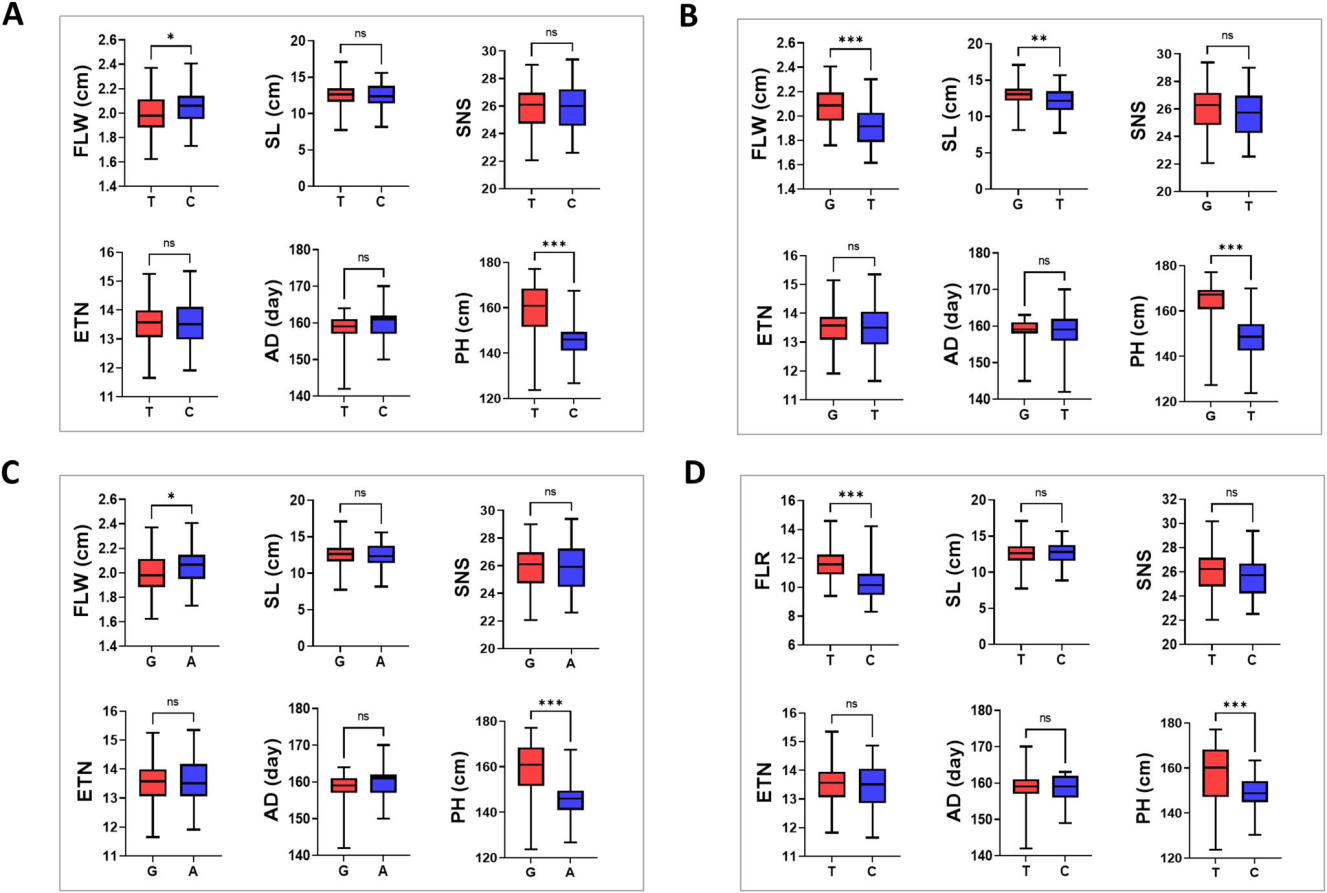
Boxplot showing the effects of *QFLW.sau.5A* (AX‐109378245, A), *QFLW.sau.5B* (AX‐110931135, B), *QFLW.sau.5D* (AX‐110979994, C), and *QFLR.sau.3D* (AX‐109854359, D) by comparing two allelic groups. AD, anthesis date; ETN, effective tiller number; FLR, flag leaf ratio; FLW, flag leaf width; PH, plant height; SL, spike length; SNS, spikelet number per spike. *, **, and *** denote significance at the 0.05, 0.01, and 0.001 probability levels, respectively; ns, non‐significant.

*QFLW.sau.5A* (AX‐109378245, T/C) showed a significant association with PH. Although the C allele contributed to wider flag leaves, lines carrying this allele were significantly shorter than those carrying the T allele, indicating a trade‐off between FLW and PH (Figure 4A). Similarly, *QFLW.sau.5D* (AX‐110979994, G/A) displayed a comparable effect: the A allele was associated with increased FLW but reduced PH (Figure 4C). Interestingly, *QFLW.sau.5B* (AX‐110931135, G/T) influenced both SL and PH in addition to FLW (Figure 4B). Lines carrying the G allele exhibited significantly wider flag leaves, longer spikes, and greater PH compared with those carrying the T allele, suggesting pleiotropic effects of this locus on multiple yield‐related traits. For *QFLR.sau.3D* (AX‐109854359, T/C), the C allele was associated with both reduced FLR and shorter PH, further supporting the role of this locus in regulating leaf architecture and plant stature (Figure 4D).

### 3.6 Validation of three potential novel QTL for FLW

**FIGURE 5:**
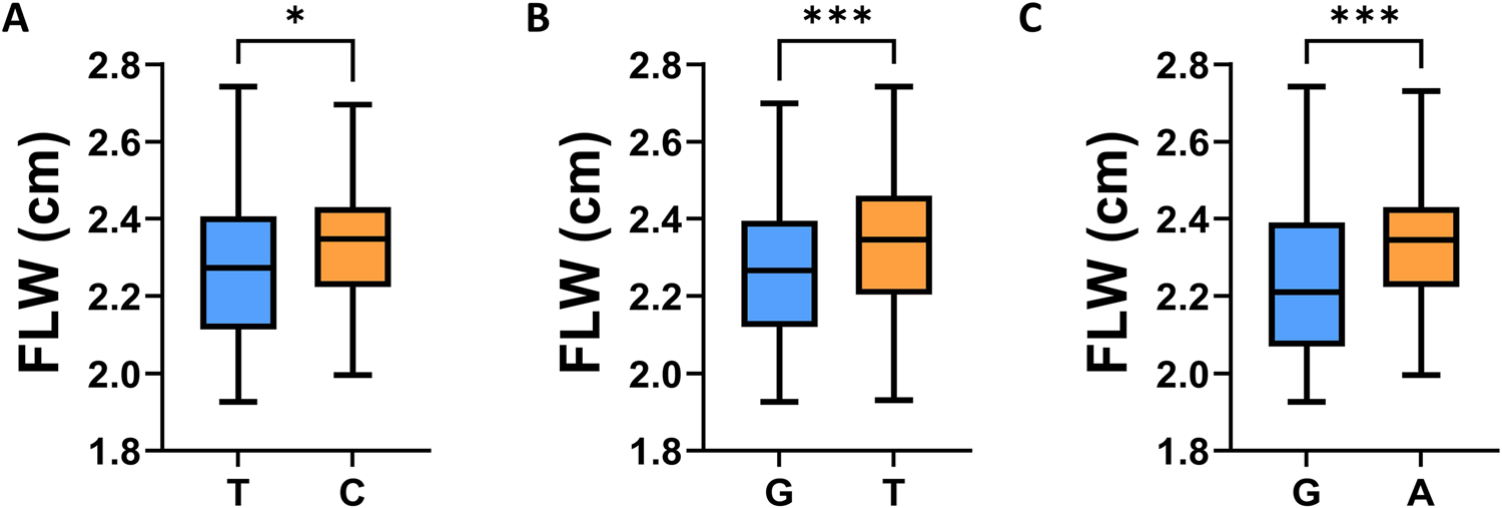
Boxplot showing the effects of *QFLW.sau.5A* (AX‐109378245, A), *QFLW.sau.5B* (AX‐110931135, B), and *QFLW.sau.5D* (AX‐110979994, C) by comparing two allelic groups in an independent natural population. FLW, flag leaf width. * and *** denote significance at the 0.05 and 0.001 probability levels, respectively.

Three potentially novel QTL for FLW identified in our study were further investigated in 220 accessions of Chinese wheat landraces and cultivars (Figure 5A–C). The validation process was conducted using genotyping data obtained from the 55K SNP array (Ye et al., 2019). A QTL was considered validated when the same SNP, or a closely linked SNP within the corresponding LD interval, showed a significant allelic effect (*p* ≤ 0.001) in the validation population, as determined by a *t*‐test.

Two of the three QTL, *QFLW.sau.5A* and *QFLW.sau.5D*, showed consistent allelic effects between the GWAS and validation populations, with favorable alleles associated with increased FLW in both panels. For the remaining QTL *QFLW.sau.5B*, although a significant allelic effect was detected (Figure 5B), the direction of effect differed between the two populations. This inconsistency may reflect differences in genetic background, allele frequency, or genotype‐by‐environment interactions.

Overall, these results support the stability of the majority of detected loci while highlighting potential context‐dependent effects for specific QTL.

### 3.7 Candidate gene prediction

Candidate gene analysis was conducted using the IWGSC RefSeq v2.1 annotation to identify genes associated with FLM traits from three FLW‐related QTLs. To identify potential candidate genes, we examined the genomic regions surrounding each QTL. Based on the LD half‐decay distance, we opted for a ±2.5 Mb window to ensure gene prioritization remained focus and biologically relevant. A total of 401 genes were initially extracted from these regions. To prioritize functionally relevant genes, transcriptome expression data (TPM values) across multiple wheat tissues were examined using wheat expression database. A total of 13 HC genes were selected based on their higher expression levels in flag leaf tissues compared to other organs (e.g., root, spike, grain), suggesting tissue‐specific activity linked to leaf growth and development (Table 4). Among these genes, six were from *QFLW.sau.5A*, three from *QFLW.sau.5B*, and four were from *QFLW.sau.5D*. Notably, some of the prioritized genes, such as the protein kinases *TraesCS5A02G241300* and *TraesCS5D02G247800*, are located in the syntenic regions of chromosomes 5A and 5D, indicating they may be homoeologous copies of the same ancestral gene. These genes are involved in different biological processes encoding protein kinase, ATP synthase subunit, protein disulfide isomerase, and so on, thus impacting development and photosynthesis. Interestingly, 12 out of 13 genes were also expressed in grain (Figure S3).

**TABLE 4:** List of predicted candidate genes, their functions, and homologs.

Gene Id	Start position (Mb)	Function	Homologs in rice and *Arabidopsis*
*TraesCS5A02G241300*	457.625916	Protein kinase	*OsRLCK118*, *OsRLCK176*, *OsRLCK107*, *APK1A*, *PK1B*, *NAK*
*TraesCS5D02G247800*	357.92695		
*TraesCS5A02G241500*	457.798513	Hfr‐2‐like protein	None
*TraesCS5A02G241700*	457.915787	Alpha/beta‐Hydrolases superfamily protein	*CXE13*, *CXE12*
*TraesCS5A02G242500*	458.252269		
*TraesCS5D02G249100*	358.777145		
*TraesCS5A02G245200*	459.196645	Transcription factor, putative (Protein of unknown function, DUF547)	None
*TraesCS5D02G251800*	360.473726		
*TraesCS5A02G245700*	459.495981	Carbonic anhydrase	*OsbetaCA2*, *BETACA1*, *BETACA5*, *BETACA6*
*TraesCS5B02G048300*	55.58793	Glutaredoxin family protein	*OsGRX4*, *OsGRX28*, *OsGRX7*
*TraesCS5B02G052700*	58.587514	ATP synthase subunit c, chloroplastic	None
*TraesCS5B02G053300*	59.261578	RING‐finger, DEAD‐like helicase, PHD, and SNF2 domain‐containing protein	None
*TraesCS5D02G245900*	357.233992	Protein disulfide‐isomerase SCO2	*OsCYO1*, *SCO2*

*Note*: GeneID and position of gene are based on IWGSC CS RefSeq v2.1.

## 4 DISCUSSION

The flag leaf plays a pivotal role in wheat productivity, and our findings provide new insights into the genetic basis of its morphology. Across 182 Chinese endemic accessions, FLM traits displayed continuous variation and high heritability, confirming polygenic control and aligning with previous studies (S. Chen et al., 2021; Kumar et al., 2024; Y. Wang, Qiao, et al., 2022). Importantly, trait correlations were consistent across environments, suggesting that genetic effects predominate, even though environmental influences remain detectable. The strong association between FLW and FLA underscores the potential of leaf width as a breeding target to enlarge photosynthetic surface area, in agreement with Zhao et al. (2022). Interestingly, FLL was positively correlated with FLW, a finding that contrasts with weaker or absent correlations reported elsewhere (Kumar et al., 2024; Schierenbeck et al., 2024), implying that the Chinese germplasm may harbor distinct genetic determinants. Consistent with K. Liu et al. (2018), FLM traits were positively linked with SL, while FLW showed particular influence on SNS, highlighting its dual role in both leaf architecture and spike development. Comparisons of FLM traits between the two subgroups inferred from population structure analysis revealed no significant differences (Figure S4), indicating minimal impact of population stratification on FLM traits and supporting the reliability of subsequent GWAS results.

GWAS detected four QTL for FLM: three for FLW on 5A, 5B, and 5D, and one for FLR on 3D. The GWAS panel used in this study consists mainly of Chinese endemic wheat, including landraces and semi‐wild accessions, which are genetically distinct from modern elite cultivars. Although the identified QTL were validated in an independent natural population, the favorable alleles are likely underrepresented in current breeding germplasm and may provide valuable genetic resources for improving flag leaf traits. Further evaluation in elite breeding backgrounds will be necessary to assess their practical utility.

To rigorously assess the novelty of the four QTL identified in this study, we compared their genomic intervals with those of previously reported loci for FLM (Table S2). Although numerous studies have reported FLW QTL, none of the loci identified here co‐localize with these previously reported regions (Fan et al., 2015; K. Liu et al., 2018; Zhao et al., 2022). Specifically, *QFLW.sau.5A* (peak at ∼459.0 Mb) is located on the long arm of chromosome 5A and is clearly distinct from previously reported loci, including *MQTL‐5A.4* (644.1–663.8 Mb; Kong et al., 2023), the QTN RAC875_rep_c112818_307 (∼613.5 Mb; Schierenbeck et al., 2024), and other meta‐QTL regions (*MQTL‐34*, *MQTL‐36*, *MQTL‐37*; Du et al., 2022). Similarly, *QFLW.sau.5B* (peak at ∼57.0 Mb) is positioned on the short arm of chromosome 5B and is physically distant from known FLW loci such as *QFlw.sau‐AM‐5B* (∼604.7–610.7 Mb; J. Wang, Liu, et al., 2022), as well as previously reported meta‐QTL (*MQTL‐38*, *MQTL‐41*; Du et al., 2022; Kong et al., 2023). The third FLW QTL, *QFLW.sau.5D* (peak at ∼358.0 Mb), is located on the long arm of chromosome 5D and does not overlap with earlier reported loci, including the major QTL on the short arm (*QFLW.5D*; Yan et al., 2020) or meta‐QTL regions on chromosome 5D (*MQTL‐42*, *MQTL‐43*; Du et al., 2022). Likewise, *QFLR.sau.3D* (peak at ∼30.2 Mb) is positioned outside previously reported regions for flag leaf traits, with the closest meta‐QTL (*MQTL‐22*) located more than 30 Mb away (Du et al., 2022). This comprehensive comparison confirms that all four QTL are distinct from previously reported loci, underscoring the unique value of the Chinese endemic wheat panel for discovering novel genetic determinants underlying FLM.

All four loci influenced PH, with *QFLW.sau.5B* additionally affecting SL, reflecting pleiotropy. Notably, the C allele of *QFLW.sau.5A* (AX‐109378245, T/C) and the A allele of *QFLW.sau.5D* (AX‐110979994, G/A) both increased FLW while reducing PH, providing an advantageous combination of enhanced photosynthetic area and shorter stature that can improve canopy efficiency and lodging resistance. These pleiotropic effects warrant consideration for breeding applications. Neither locus coincides with known *Rht* dwarfing genes, which are located on chromosomes 2D (*Rht8*), 4B and 4D (*Rht‐B1*/*D1*), and 6A (*Rht24*) (Peng et al., 1999; Tian et al., 2022; Xiong et al., 2022), suggesting these QTL represent novel height‐regulating mechanisms. The observed trade‐off between FLW and PH may be addressed through fine‐mapping to determine whether the pleiotropy results from a single gene or tightly linked genes. If due to linkage, recombination could separate the favorable FLW allele from the height‐reducing allele. Alternatively, screening for additional haplotypes that confer increased FLW without height penalties could provide more favorable alleles for breeding programs. Validation of three FLW QTL in an independent population confirms their stability and breeding value, making these loci strong candidates for developing high‐yield wheat ideotypes with optimized leaf architecture and improved spike performance.

Notably, the three novel FLW QTL were mapped to the homoeologous group‐5 chromosomes (5A, 5B, and 5D), suggesting the presence of a conserved genetic system inherited from a common ancestral genome. The clustering of phenotypically related QTL on homoeologous chromosomes is a well‐documented phenomenon in polyploid wheat and often points to the coordinated action of homoeologous gene copies in regulating complex traits (Ramírez‐González et al., 2018). In this study, the co‐localization of FLW QTL suggests that the underlying homoeologous genes in these syntenic regions have been preserved across the A, B, and D subgenomes to collectively modulate flag leaf development. This genomic arrangement provides a basis for genetic redundancy and buffering, and may allow for fine‐tuning of the trait through allelic variation at each homoeologous locus.

From three novel FLW QTL, 13 candidate genes were identified, highlighting diverse pathways influencing flag leaf development. Protein kinases *TraesCS5A02G241300* and *TraesCS5D02G247800*, with rice (*Oryza sativa*) orthologs *OsRLCK118*, *OsRLCK176*, and *OsRLCK107*, likely regulate growth via brassinosteroid signaling (X. Zhou et al., 2016). Their presence as candidate genes in two of the three homoeologous FLW QTL regions supports the model of a conserved, multi‐copy genetic module regulating this trait. Additional kinase‐related genes, including *APK1A*, *PK1B*, and *NAK*, may modulate signal transduction and phosphorylation critical for leaf expansion.

Beyond regulatory and signaling components, several candidate genes encode proteins with direct roles in photosynthetic metabolism and chloroplast function. *TraesCS5A02G245700* encodes a carbonic anhydrase, which facilitates CO_2_ hydration and is essential for normal photosynthetic carbon assimilation and plant growth (Weerasooriya et al., 2022). Complementing this role in carbon fixation, *TraesCS5B02G052700* (chloroplast ATP synthase subunit C) and *TraesCS5D02G245900* (protein disulfide‐isomerase SCO2) contribute to chloroplast biogenesis and energy metabolism. ATP synthase is critical for generating the ATP required for carbon fixation, while SCO2 functions in chloroplast development and photosynthetic complex assembly (Zagari et al., 2017). Collectively, these genes support the photosynthetic capacity of the flag leaf, thereby influencing the assimilate supply available for grain filling—a key determinant of yield.

*TraesCS5B02G053300*, a multi‐domain protein containing RING‐finger, DEAD‐like helicase, PHD, and SNF2 domains, may influence leaf development via chromatin remodeling, protein turnover, and chloroplast translation (X. Y. Li et al., 2011; Nawaz & Kang, 2019; Shu & Yang, 2017). Transcription factors *TraesCS5A02G245200* and *TraesCS5D02G251800*, α/β‐hydrolases *TraesCS5A02G241700*, *TraesCS5A02G242500*, *TraesCS5D02G249100*, and redox proteins *TraesCS5A02G241500* and *TraesCS5B02G048300* likely contribute to growth, photosynthesis, and stress adaptation (Rui et al., 2022; Zhai et al., 2023). Collectively, these genes integrate signaling, energy metabolism, transcriptional regulation, and stress resilience pathways, shaping FLM, maintaining photosynthetic capacity, and ultimately supporting grain yield.

It is important to note that the phenotypic variance explained by individual QTL was modest, consistent with the polygenic architecture of flag leaf traits, and indicates that these loci likely function alongside additional genetic factors. Furthermore, while our candidate gene predictions were informed by LD patterns, tissue‐specific expression, and functional annotations, they remain putative until validated experimentally. Future work should therefore prioritize fine‐mapping of the QTL intervals, expression profiling via quantitative Reverse Transcription PCR in near‐isogenic or contrasting genotypes, and functional characterization using transgenic or gene‐editing approaches. Although marker–trait associations were validated in an independent population, their evaluation across a broader range of environments and genetic backgrounds will be essential to confirm stability and breeding utility. The observed pleiotropy between flag leaf traits and PH also underscores the need for balanced selection in breeding programs to optimize canopy architecture without compromising agronomic performance.

In summary, this integrative approach combining GWAS, LD‐based region definition, tissue‐specific expression profiling, and functional annotation provides a robust framework for identifying candidate genes underlying complex traits like FLM. These findings offer actionable insights into wheat breeding programs targeting leaf architecture and associated physiological traits.

## 5 CONCLUSION

This study evaluated 182 genetically diverse accessions of Chinese endemic wheat, measuring key flag leaf traits such as length (FLL), width (FLW), area (FLA), and ratio (FLR). By analyzing 38,490 HC molecular markers through GWAS, four new candidate loci were identified. Further, 13 candidate genes for FLM were predicted based on the expression level in flag leaf tissue compared to others. These results provide useful genetic markers and elite germplasm for future breeding programs aimed at crop improvement.

## AUTHOR CONTRIBUTIONS

**Md Nahibuzzaman Lohani**: Formal analysis; investigation; methodology; resources; software; validation; visualization; writing—original draft. **Longxing Su**: Data curation; formal analysis; investigation; resources; visualization; writing—original draft. **Lu Lu**: Data curation; formal analysis; investigation. **Li Yin**: Formal analysis; investigation; resources. **Yanlin Liu**: Data curation; formal analysis; investigation; resources. **Qiang Xu**: Data curation; formal analysis. **Yunfeng Jiang**: Data curation; formal analysis. **Qiantao Jiang**: Data curation; formal analysis. **Guoyue Chen**: Data curation; formal analysis. **Yuming Wei**: Data curation; formal analysis. **Chunji Liu**: Data curation; formal analysis. **Quan Xie**: Data curation; formal analysis; methodology; resources; writing—review and editing. **Jian Ma**: Conceptualization; formal analysis; funding acquisition; investigation; project administration; resources; supervision; writing—review and editing.

## CONFLICT OF INTEREST STATEMENT

All authors declare no conflicts of interest.

## Supporting information




**Figure S1**. Population Structure Inferred by STRUCTURE, PCA Analysis and LD half decay. **A,** Delta K values plotted against the number of clusters (K). The highest peak at K = 2 indicates the most likely number of genetic clusters in 182 accessions of Chinese endemic wheat. **B**, Three‐dimensional principal component analysis (PCA) plots of individual genotypes. **C**, LD half decay distance. **D**, STRUCTURE bar plot showing population structure at K = 2. **E**, Kinship matrix heatmap representing the genetic relatedness among accessions.
**Figure S2**. Manhattan and quantile‐quantile (Q‐Q) plots showing the results of genome wide association studies (GWAS) for **A**; FLW, flag leaf width; **B**; FLR, flag leaf ratio using Q+K, MLM method. The positions of SNP on chromosomes are indicated on the x‐axis relative to their ‐log_10_(*P*) values on the y‐axis. The red dotted line refers to the threshold of ‐log_10_(*P*) = 3.
**Figure S3**. Expression profiles of predicted genes across wheat grain developmental stages.
**Figure S4**. Comparison between two sub‐groups for FLM traits across different environments (2022CZ,2023CZ,2025CZ and 2025WJ). FLL, flag leaf length; FLW, flag leaf width; FLA, flag leaf area; FLR, flag leaf ratio.

**Table S1**. The information about the 182 accessions of Chinese endemic wheat.
**Table S2**. Comparison between the previously detected QTL with identified QTL in this study.

## Data Availability

The phenotypic data for all environments (2022CZ, 2023CZ, 2025CZ, and 2025WJ), together with the corresponding best linear unbiased prediction (BLUP) values, have been deposited in Figshare under accession https://doi.org/10.6084/m9.figshare.30929660. The genotypic dataset, consisting of the filtered 38,490 SNP matrix for the 182 Chinese endemic wheat accessions, is available in the same repository under the same accession. In addition, the lists of predicted candidate genes within the intervals of the three newly identified QTL for flag leaf width (FLW), as well as the corresponding gene expression data, are also provided in this repository. All other data generated or analyzed during this study are included in this published article and its Supporting Information. Further inquiries may be directed to the corresponding authors.
