# Supplementary material for: Genetic identification and characterization of novel loci for flag leaf morphology traits in Chinese endemic wheat
Source: Plant Genome. 2026 Apr 28;19(2):e70245. doi: 10.1002/tpg2.70245 (PMC13122275; doi:10.1002/tpg2.70245)
Supplement: Supplementary file 1 [file TPG2-19-e70245-s001.pdf]

Figure S1

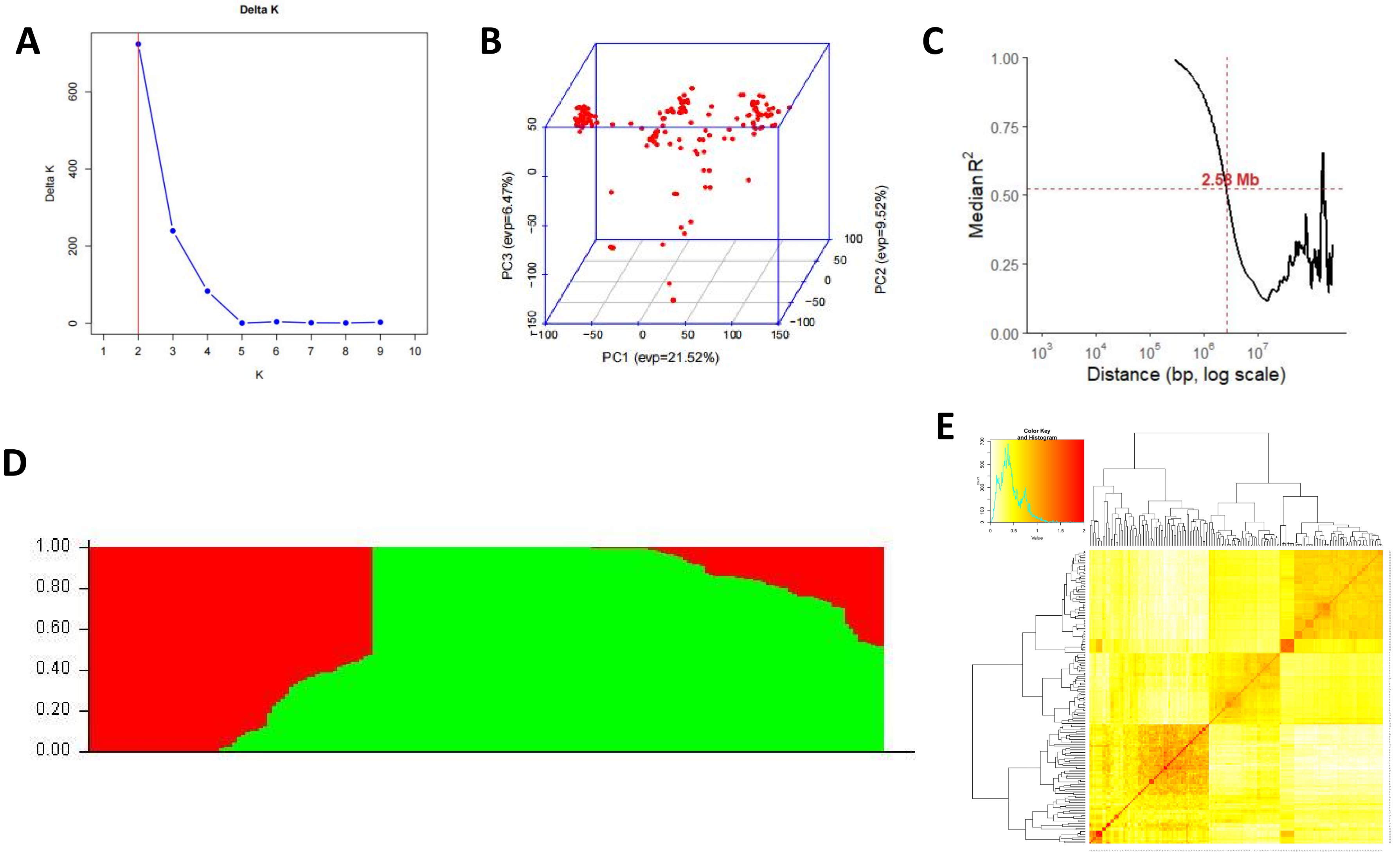
A
Delta K
Delta K
0
200
400
600
1
2
3
4
5
6
7
8
9
10
K
B
PC1 (evp=21.52%)
PC2 (evp=9.52%)
PC3 (evp=6.47%)
−100
−50
0
50
100
150
C
Median R^2
Distance (bp, log scale)
2.53 Mb
0.00
0.25
0.50
0.75
1.00
10^3
10^4
10^5
10^6
10^7
D
1.00
0.80
0.60
0.40
0.20
0.00
E
Color Key and Histogram
Value

Figure S2

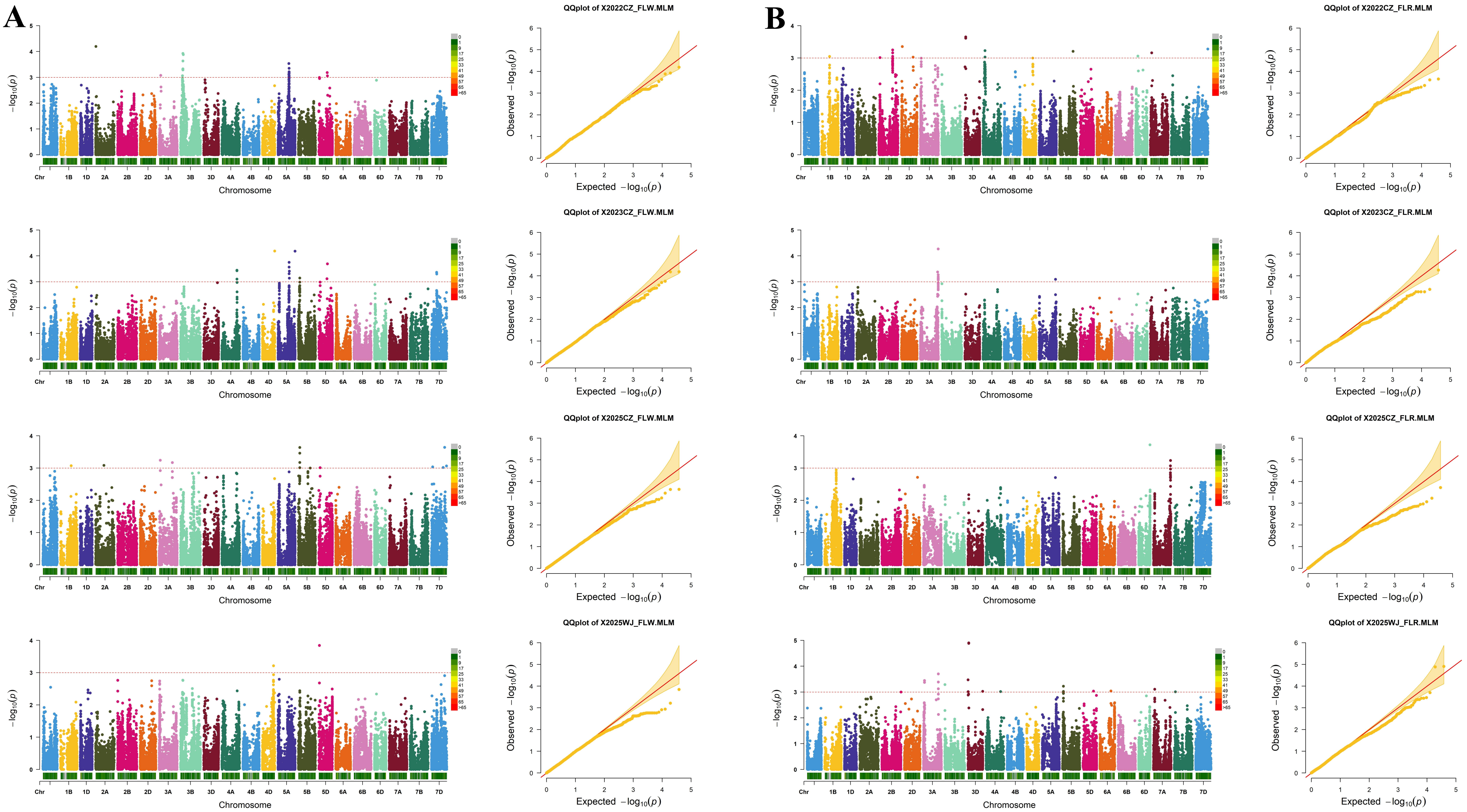
A
B
QQplot of X2022CZ_FLW.MLM
QQplot of X2023CZ_FLW.MLM
QQplot of X2025CZ_FLW.MLM
QQplot of X2025WJ_FLW.MLM
QQplot of X2022CZ_FLR.MLM
QQplot of X2023CZ_FLR.MLM
QQplot of X2025CZ_FLR.MLM
QQplot of X2025WJ_FLR.MLM
$-\log_{10}(p)$
Chromosome
Chr 1B 1D 2A 2B 2D 3A 3B 3D 4A 4B 4D 5A 5B 5D 6A 6B 6D 7A 7B 7D
0 1 9 17 25 33 41 49 57 65 >65
Observed $-\log_{10}(p)$
Expected $-\log_{10}(p)$

Figure S3

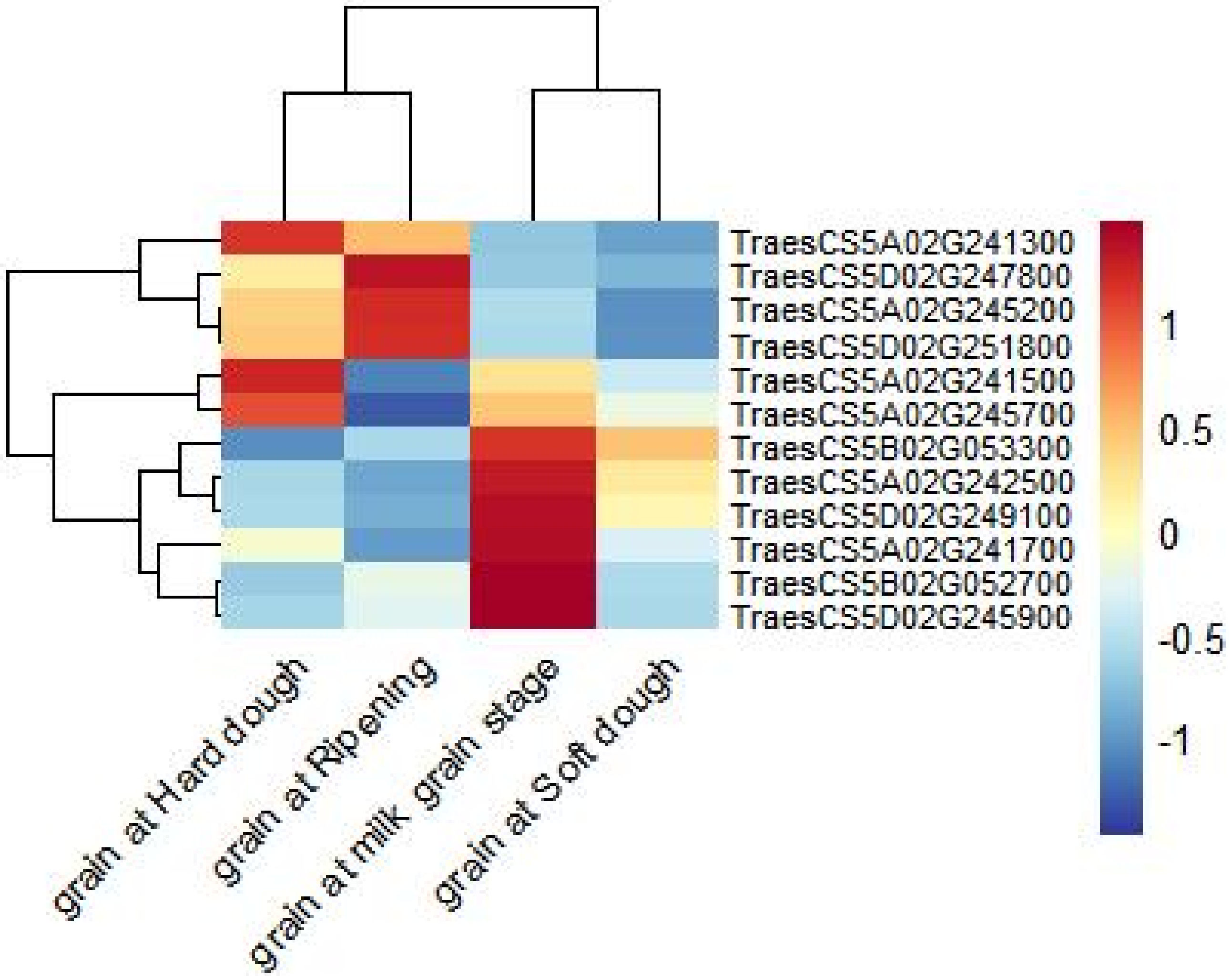
TraesCS5A02G241300
TraesCS5D02G247800
TraesCS5A02G245200
TraesCS5D02G251800
TraesCS5A02G241500
TraesCS5A02G245700
TraesCS5B02G053300
TraesCS5A02G242500
TraesCS5D02G249100
TraesCS5A02G241700
TraesCS5B02G052700
TraesCS5D02G245900
grain at Hard dough
grain at Ripening
grain at milk grain stage
grain at Soft dough
1
0.5
0
-0.5
-1

Figure S4

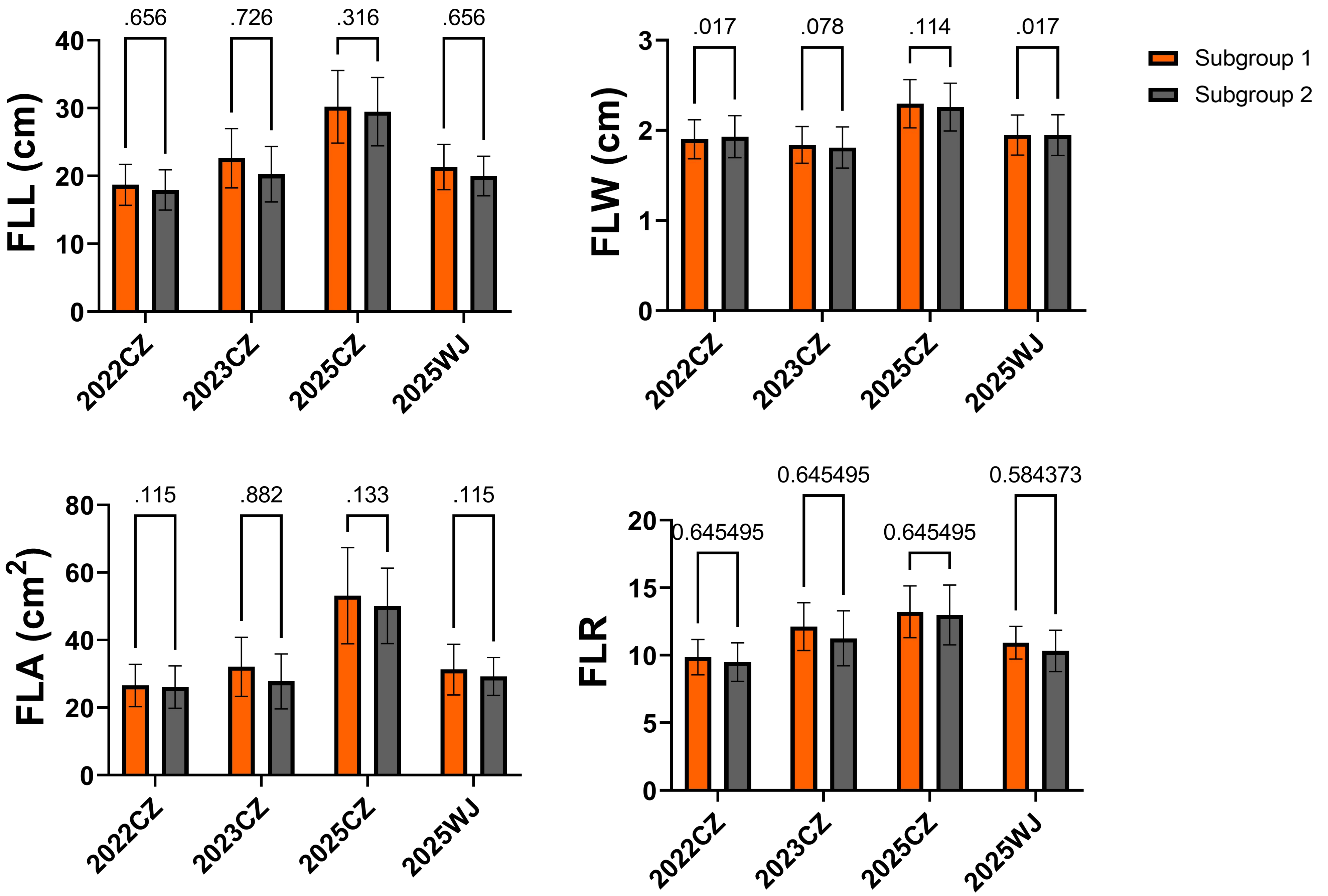

FLL (cm)
FLW (cm)
FLA (cm²)
FLR
2022CZ
2023CZ
2025CZ
2025WJ
.656
.726
.316
.656
.017
.078
.114
.017
.115
.882
.133
.115
0.645495
0.645495
0.645495
0.584373
Subgroup 1
Subgroup 2
