# Supplementary figures and images for: Genetic identification and characterization of novel loci for flag leaf morphology traits in Chinese endemic wheat
Source: Plant Genome. 2026 Apr 28;19:e70245. doi: 10.1002/tpg2.70245 (PMC13122275; doi:10.1002/tpg2.70245)

Figure S1

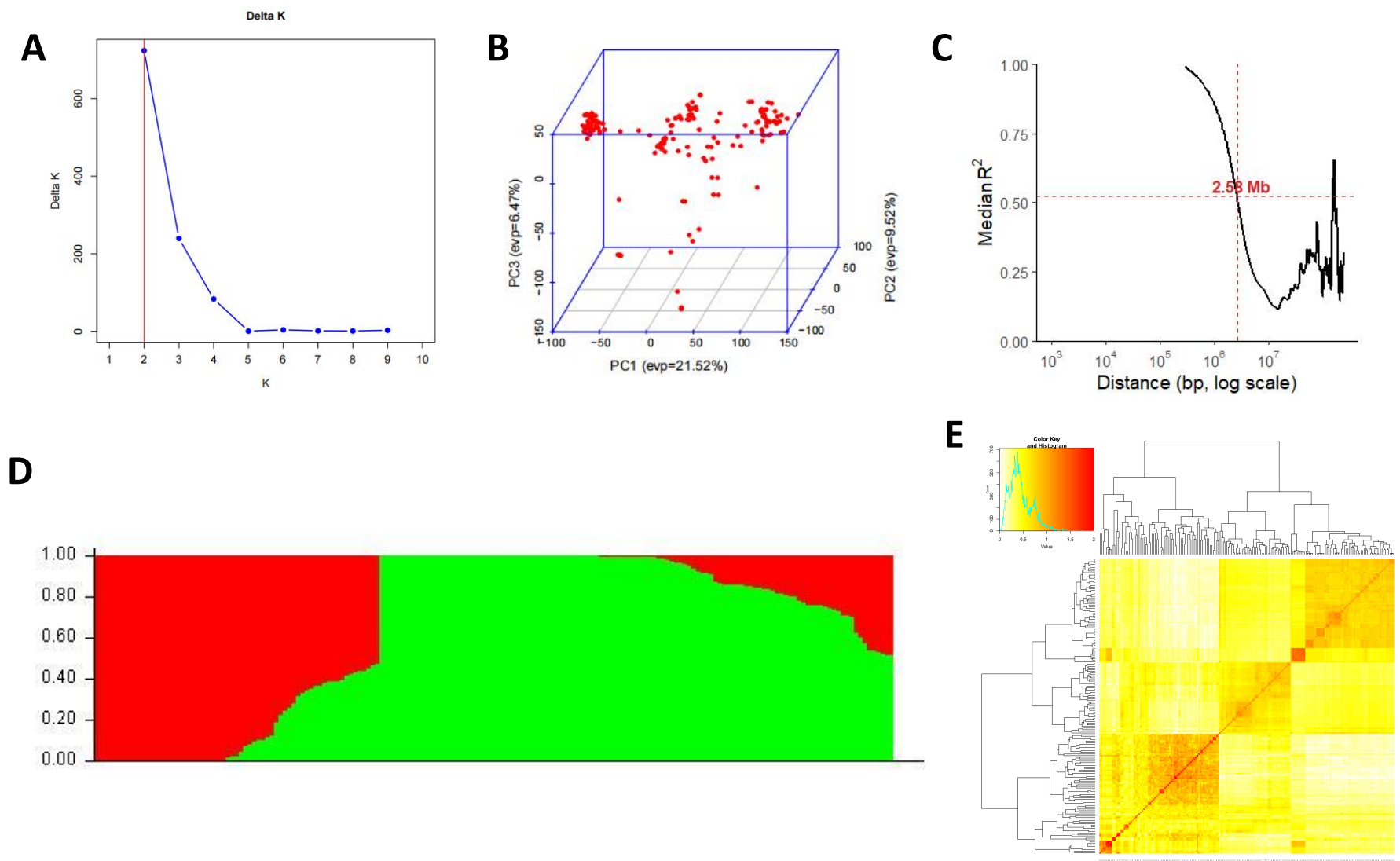

Figure S2

A

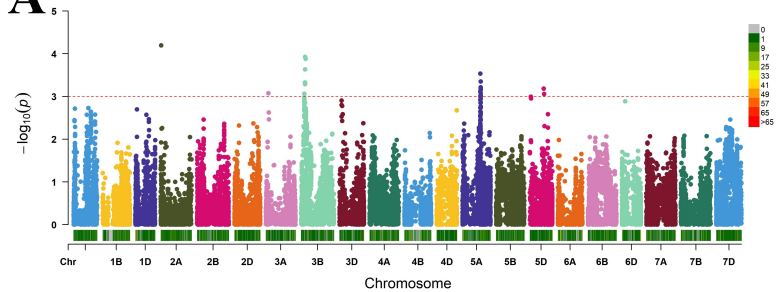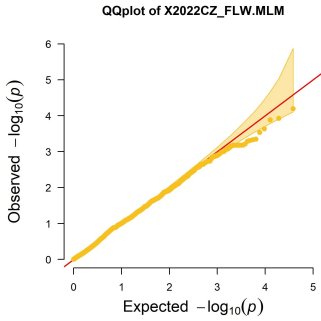

B

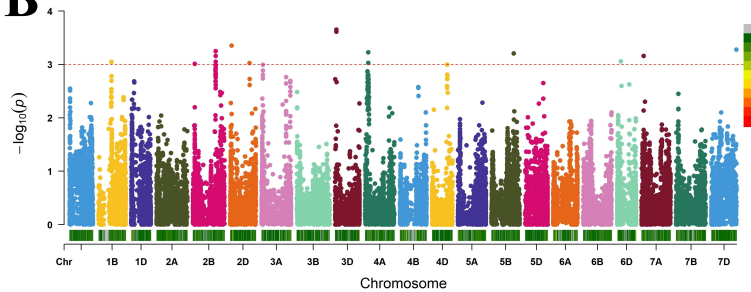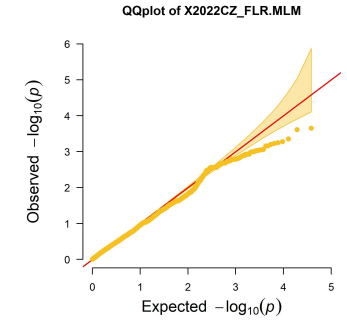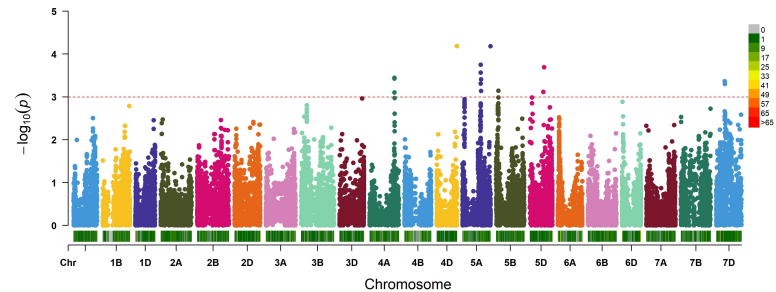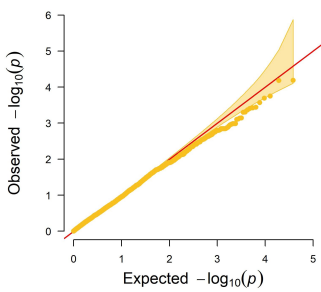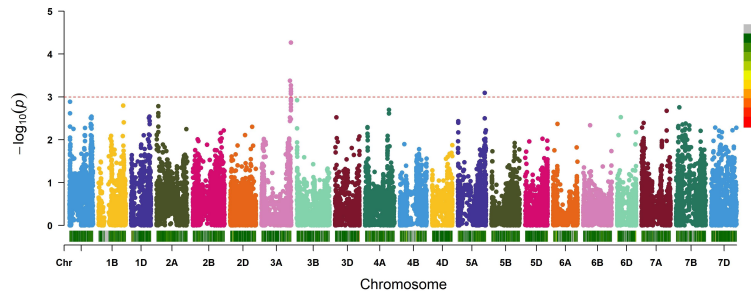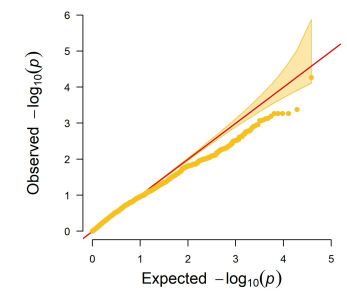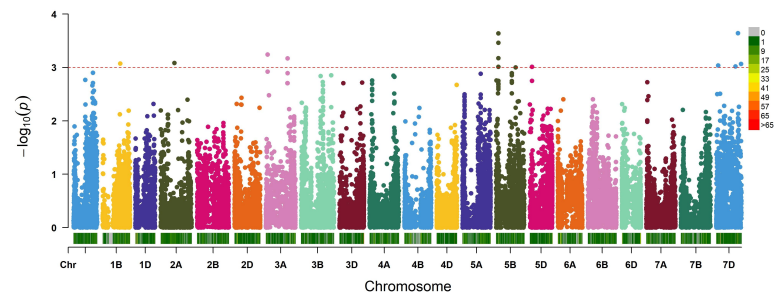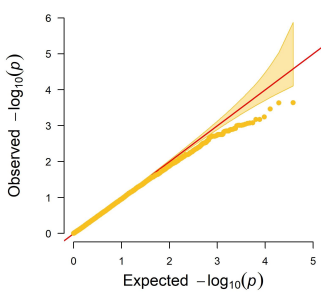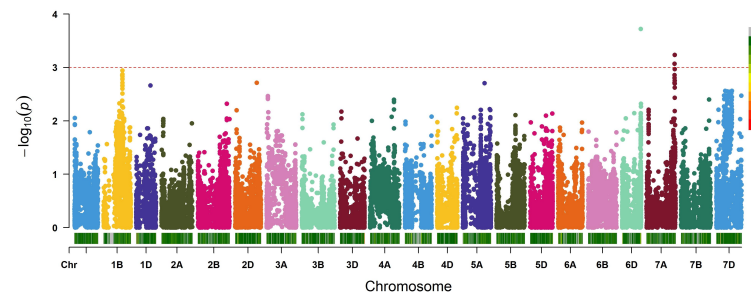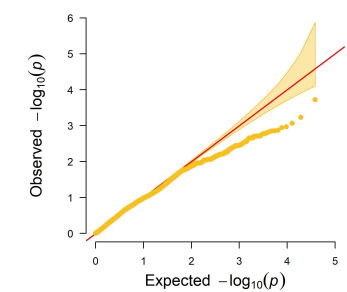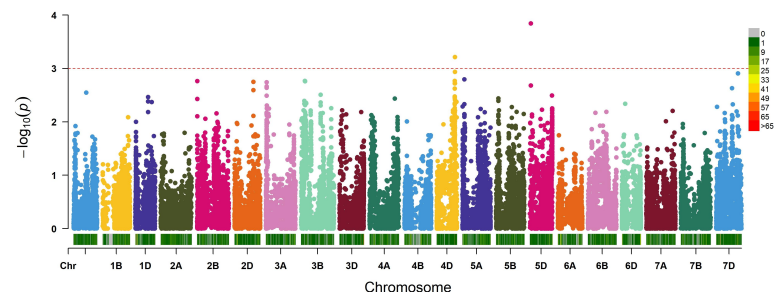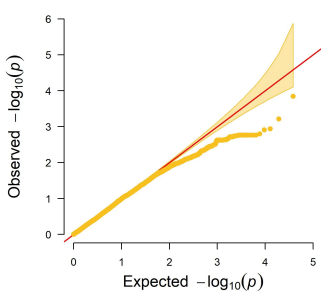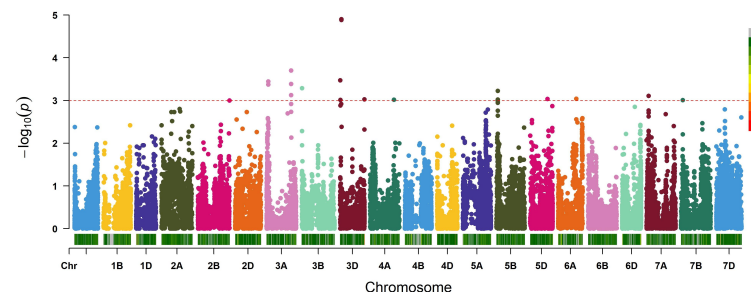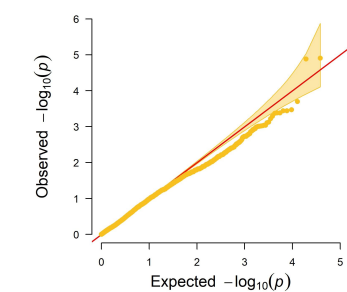

Figure S3

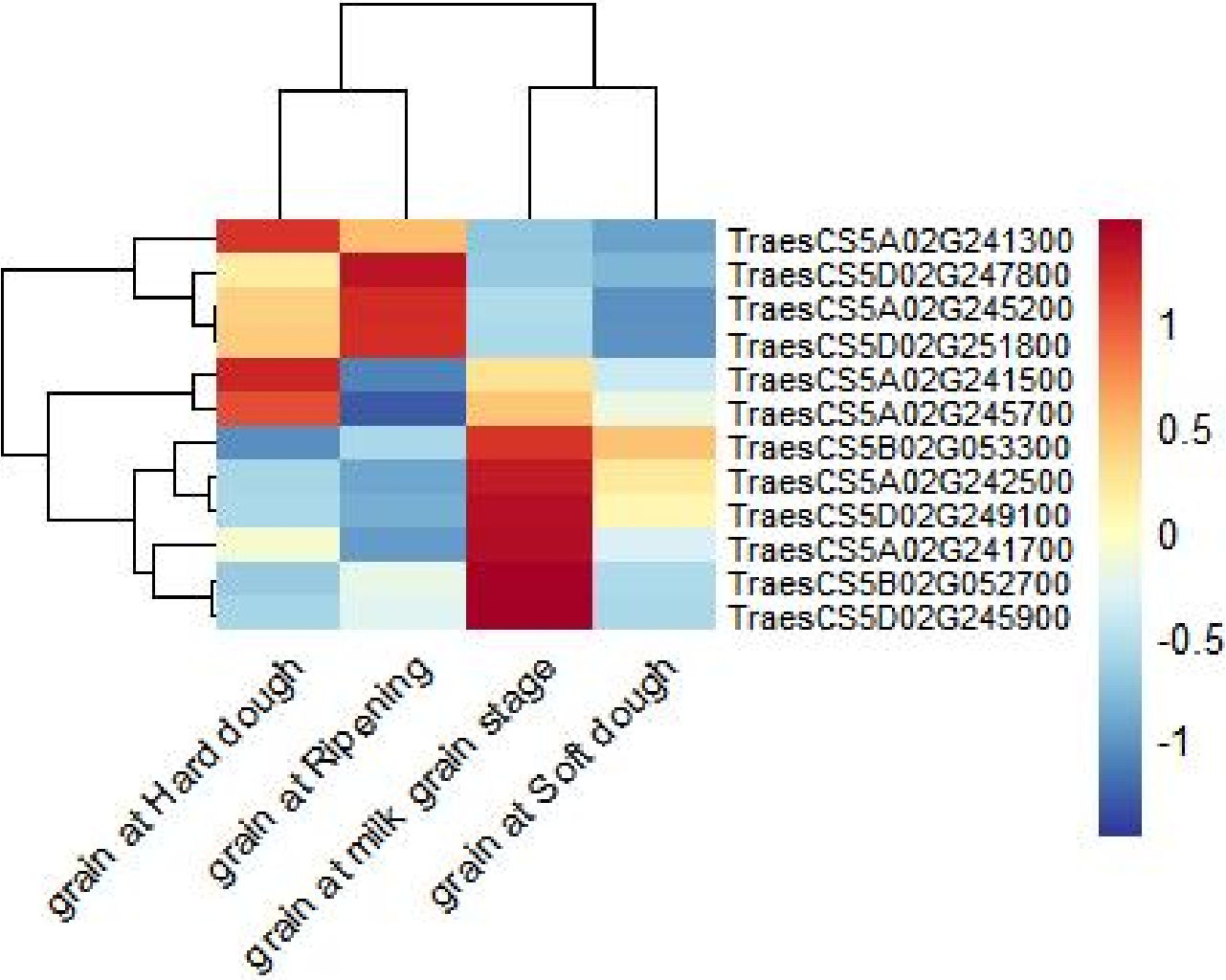

Figure S4

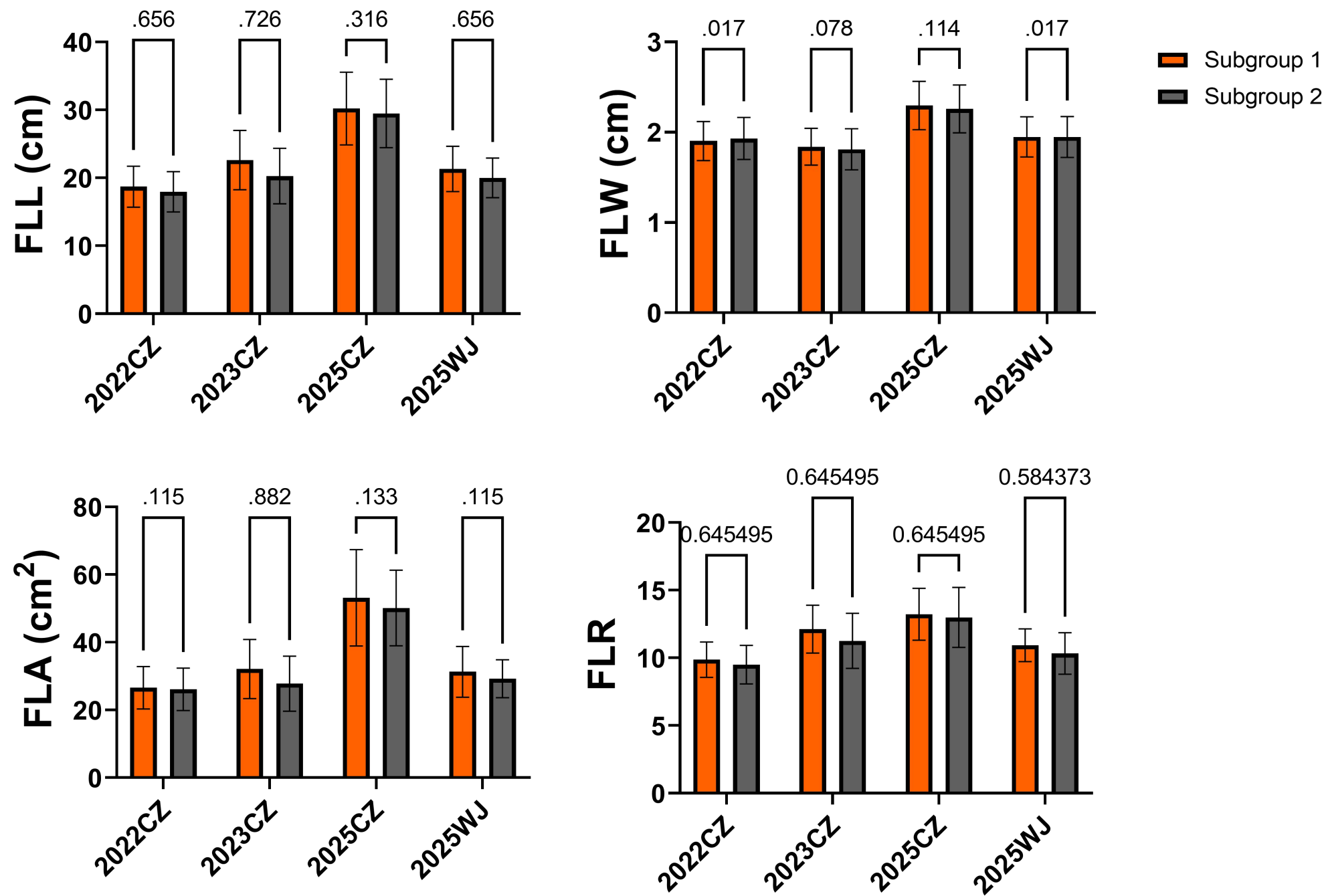

Supplement: Supplementary file 1 — Figure S1. Population Structure Inferred by STRUCTURE, PCA Analysis and LD half decay. A, Delta K values plotted against the number of clusters (K). The highest peak at K = 2 indicates the most likely number of genetic clusters in 182 accessions of Chinese endemic wheat. B, Three‐dimensional principal component analysis (PCA) plots of individual genotypes. C, LD half decay distance. D, STRUCTURE bar plot showing population structure at K = 2. E, Kinship matrix heatmap representing the genetic relatedness among accessions. Figure S2. Manhattan and quantile‐quantile (Q‐Q) plots showing the results of genome wide association studies (GWAS) for A; FLW, flag leaf width; B; FLR, flag leaf ratio using Q+K, MLM method. The positions of SNP on chromosomes are indicated on the x‐axis relative to their ‐log10(P) values on the y‐axis. The red dotted line refers to the threshold of ‐log10(P) = 3. Figure S3. Expression profiles of predicted genes across wheat grain developmental stages. Figure S4. Comparison between two sub‐groups for FLM traits across different environments (2022CZ,2023CZ,2025CZ and 2025WJ). FLL, flag leaf length; FLW, flag leaf width; FLA, flag leaf area; FLR, flag leaf ratio. [file TPG2-19-e70245-s001.pdf]
