# Supplementary material for: Genetic identification and characterization of novel loci for flag leaf morphology traits in Chinese endemic wheat
Source: Plant Genome. 2026 Apr 28;19:e70245. doi: 10.1002/tpg2.70245 (PMC13122275; doi:10.1002/tpg2.70245)
Supplement: Supplementary file 2 — Table S1. The information about the 182 accessions of Chinese endemic wheat. Table S2. Comparison between the previously detected QTL with identified QTL in this study. [file TPG2-19-e70245-s002.pdf]

Table S1 182 accessions of Chinese endemic wheat used in this study

No.	Name	Subspecies①	Subgroup②
1	Zuogongsuisuixiaomai1	Tibetan	1
2	Zuogongsuisuixiaomai2	Tibetan	1
3	Zuogongsuisuixiaomai8	Tibetan	1
4	Jiachasuisuixiaomai220	Tibetan	1
5	Jiachasuisuixiaomai21	Tibetan	2
6	Jiachasuisuixiaomai23	Tibetan	1
7	Jiachasuisuixiaomai24	Tibetan	2
8	Jiachasuisuixiaomai27	Tibetan	1
9	Jiachasuisuixiaomai28	Tibetan	1
11	Jiachasuisuixiaomai32	Tibetan	2
12	Jiachasuisuixiaomai33	Tibetan	2
13	Jiachasuisuixiaomai34	Tibetan	2
15	Jiachasuisuixiaomai39	Tibetan	1
16	Jiachasuisuixiaomai40	Tibetan	2
17	Sangrisuisuixiaomai2	Tibetan	1
18	Sangrisuisuixiaomai4	Tibetan	1
19	Sangrisuisuixiaomai7	Tibetan	1
20	Sangrisuisuixiaomai10	Tibetan	2
21	Sangrisuisuixiaomai11	Tibetan	2
22	Langxiansuisuixiaomai5	Tibetan	2
23	Langxiansuisuixiaomai7	Tibetan	2
24	Langxiansuisuixiaomai9	Tibetan	2
25	Langxiansuisuixiaomai13	Tibetan	1
26	Langxiansuisuixiaomai14	Tibetan	1
27	Langxiansuisuixiaomai15	Tibetan	1
28	Langxiansuisuixiaomai17	Tibetan	1
29	Langxiansuisuixiaomai19	Tibetan	2
30	Langxiansuisuixiaomai20	Tibetan	2
31	Langxiansuisuixiaomai21	Tibetan	1
32	Longzisuisuixiaomai2	Tibetan	1
33	Longzisuisuixiaomai10	Tibetan	2
34	Longzisuisuixiaomai11	Tibetan	2
35	Longzisuisuixiaomai17	Tibetan	2
36	Longzisuisuixiaomai20	Tibetan	1
37	Longzisuisuixiaomai22	Tibetan	2
38	Longzisuisuixiaomai23	Tibetan	2
40	Longzisuisuixiaomai28	Tibetan	2
42	Chayusuisuimai5	Tibetan	1
43	Chayusuisuimai20	Tibetan	1
44	Chayusuisuimai27	Tibetan	1
45	Chayusuisuimai30	Tibetan	1
46	Chayusuisuimai31	Tibetan	1
47	Chayusuisuimai35	Tibetan	2
48	Chayusuisuimai40	Tibetan	1

49	Chayusuisuimai45	Tibetan	1
50	Chayazheda5	Tibetan	2
51	Chayazheda12	Tibetan	1
52	Chayazheda14	Tibetan	2
53	Chayazheda18	Tibetan	1
54	Chayazheda19	Tibetan	1
56	Chayazheda32	Tibetan	2
57	Chayazheda43	Tibetan	1
58	Chayazheda49	Tibetan	1
59	Chayazheda50	Tibetan	1
60	Chayazheda57	Tibetan	2
61	Chayuzheda5	Tibetan	1
62	Jiachazheda9	Tibetan	2
63	Jiachazheda15	Tibetan	1
64	Jiachazheda19	Tibetan	2
65	Jiachazheda27	Tibetan	1
66	Jiachazheda28	Tibetan	2
67	Jiachazheda30	Tibetan	1
68	Jiachazheda33	Tibetan	1
69	Jiachazheda37	Tibetan	1
70	Jiachazheda38	Tibetan	1
72	Jiachazheda41	Tibetan	1
73	Jiachazheda55	Tibetan	2
74	Jiachazheda63	Tibetan	2
76	Jiachazheda76	Tibetan	1
77	Langxianzheda3	Tibetan	2
78	Langxianzheda4	Tibetan	2
79	Langxianzheda10	Tibetan	2
80	Langxianzheda12	Tibetan	1
81	Langxianzheda15	Tibetan	1
82	Langxianzheda16	Tibetan	2
83	Langxianzheda20	Tibetan	1
84	Langxianzheda24	Tibetan	2
85	Langxianzheda25	Tibetan	2
86	Langxianzheda26	Tibetan	2
87	Langxianzheda31	Tibetan	1
88	Langxianzheda35	Tibetan	1
89	Langxianzheda47	Tibetan	2
90	Longzizheda2	Tibetan	2
91	Longzizheda4	Tibetan	2
92	Longzizheda9	Tibetan	2
93	Longzizheda11	Tibetan	2
94	Longzizheda12	Tibetan	2
95	Longzizheda16	Tibetan	2
96	Longzizheda19	Tibetan	2
97	Longzizheda21	Tibetan	2

98	Longzizheda37	Tibetan	2
99	Longzizheda30	Tibetan	2
100	Longzizheda32	Tibetan	2
101	Longzizheda38	Tibetan	2
102	Longzizheda56	Tibetan	1
103	Longzizheda62	Tibetan	2
104	Sangrizheda1	Tibetan	2
105	Sangrizheda16	Tibetan	2
106	Sangrizheda19	Tibetan	2
107	Sangrizheda21	Tibetan	2
109	Bibamisui	Tibetan	1
110	Chayumisui	Tibetan	1
111	Karemisui1	Tibetan	2
112	Jitangmisui1	Tibetan	1
113	Jitangmisui2	Tibetan	1
114	Jiachamisui	Tibetan	1
115	Kailangmisui	Tibetan	2
116	Xizangbanyeshengxiaomai1	Tibetan	1
117	Xizangbanyeshengxiaomai2	Tibetan	1
118	Yunxiantiekemai1	Yunnan	1
119	Shuangjiangchangmangtiekemai	Yunnan	1
120	Fengqingtiekemai	Yunnan	1
121	Yongdechangdingmangtiekemai	Yunnan	1
122	Yongdeduanmangtiekemai	Yunnan	1
123	Lancangtiekemai1	Yunnan	1
124	Lincangtiekemai1	Yunnan	1
125	Gengmatiekemai1	Yunnan	1
126	Zhenkangdingmangtiekemai	Yunnan	1
127	Tengchongyoumangtiekemai	Yunnan	1
128	Guangtoutiekemai1	Yunnan	1
129	Changmangtiekemai1	Yunnan	1
130	Guangtoumaoketiekemai	Yunnan	1
131	Changmangtiekemai2	Yunnan	1
132	Hongkeguangtoutiekemai	Yunnan	1
133	Yongdetieke	Yunnan	2
134	Baikeheibiantiekemai	Yunnan	1
135	Guangtoutiekemai2	Yunnan	1
136	Luotongtiekemai	Yunnan	1
137	Yunnantiekemai1	Yunnan	1
138	Yunnantiekemai2	Yunnan	1
139	Yunnantiekemai3	Yunnan	1
140	Yunnantiekemai4	Yunnan	1

141	Yunnantiekemai5	Yunnan	1
142	Yunnantiekemai6	Yunnan	2
143	Yunnantiekemai7	Yunnan	2
144	Yunnantiekemai8	Yunnan	1
145	Yunnantiekemai9	Yunnan	1
146	Yunnantiekemai10	Yunnan	1
147	Yunnantiekemai11	Yunnan	1
148	Yunnantiekemai12	Yunnan	1
150	Tiekemai12	Yunnan	1
151	Fengqingyingkemai1	Yunnan	1
152	Fengqingyingkemai2	Yunnan	1
154	Gengmatiekemai3	Yunnan	1
155	Lancangtiekemai2	Yunnan	1
156	Lancangtiekemai3	Yunnan	1
158	Tiekemai11	Yunnan	1
159	Lincangtiekemai2	Yunnan	1
160	Lincangtiekemai3	Yunnan	1
161	Lincangtiekemai4	Yunnan	1
162	Lincangtiekemai5	Yunnan	1
163	Changmangdahetouxiaomai	Yunnan	1
164	Dingmangdahetouxiaomai	Yunnan	1
165	Tiekemai1	Yunnan	1
166	Tiekemai2	Yunnan	1
167	Tiekemai3	Yunnan	1
168	Tiekemai4	Yunnan	1
171	Tiekemai7	Yunnan	1
172	Changmangtiekemai3	Yunnan	1
173	Shuangjiangtiekemai1	Yunnan	1
175	Tiekemai8	Yunnan	1
176	Duanmangtiekemai	Yunnan	1
177	Dingmangtiekemai1	Yunnan	1
182	Tiekemai9	Yunnan	1
185	Tiekemai13	Yunnan	1
186	Changmangyingkemai	Yunnan	1
187	Wumangyingkemai	Yunnan	1
188	Yunxiantiekemai	Yunnan	1
189	Yunxianyingkemaimai1	Yunnan	1
195	Zhenkangtiekemai3	Yunnan	1
196	Daomaizi	Xinjiang	2
197	Xizangmai(Daomaizi)1	Xinjiang	2
198	Xizangmai(Daomaizi)2	Xinjiang	2
199	Sudaomai	Xinjiang	1
200	Akesudaomai	Xinjiang	2
201	Yutiandaomai	Xinjiang	2
202	Luopudaomai	Xinjiang	2

204	Xinjiangdaomai1	Xinjiang	2
210	Xinjiangdaomai7	Xinjiang	1
211	Xinjiangdaomai8	Xinjiang	1
212	Xinjiangdaomai9	Xinjiang	1
213	Xinjiangdaomai10	Xinjiang	1

① The subspecies of each accession, Tibetan represent Tibetan semi-wild wheat, Yunnan represent Yunnan hulled wheat, Xinjiang represent Xinjiang rice wheat;

② Grouping by genotypic data using STRUCTURE v2.3.4.

Table S2 Comparison between the previously detected QTL with identified QTL in this study.

QTL Name (This Study)	Peak Position (Mb)	Interval (Mb)	Previously Reported QTL/QTN	Reported Position/Interval	Reference	Overlap?
QFLW.sau.5A	458.996188	456.5–461.5	*MQTL-5A.4*	644.1-663.8 Mb	Kong et al. 2023	No
			RAC875_rep_c112818_307	613.5 Mb	Schierenbeck et al. 2024	No
			MQTL-34	253.6-306.5 Mb	Du et al. 2022	No
			MQTL-36	555.2-594.1 Mb	Du et al. 2022	No
			MQTL-37	680.5-681.9 Mb	Du et al. 2022	No
QFLW.sau.5B	56.954562	54.5–59.5	*QFlw.sau-AM-5B*	604.7- 610.7 Mb	Wang et al. 2022	No
			MQTL-38	355.2-437.8 Mb	Du et al. 2022	No
			MQTL-41	678.0-678.6 Mb	Du et al. 2022	No
			MQTL-5B.3	670.5-690.2 Mb	Kong et al. 2023	No
QFLW.sau.5D	358.012495	355.5–360.5	*QFLW.5D*	5–12 cM (5DS)	Yan et al. 2020	No
			MQTL-42	58.1-235.5 Mb	Du et al. 2022	No
			MQTL-43	401.4-411.2 Mb	Du et al. 2022	No
QFLR.sau.3D	30.233478	27.73 – 32.73	*MQTL-22*	62.1-179.3 Mb	Du et al. 2022	No
